# The origin of Neolithic copper on the central Northern European plain and in Southern Scandinavia: Connectivities on a European scale

**DOI:** 10.1371/journal.pone.0283007

**Published:** 2023-05-10

**Authors:** Jan Piet Brozio, Zofia Stos-Gale, Johannes Müller, Nils Müller-Scheeßel, Sebastian Schultrich, Barbara Fritsch, Fritz Jürgens, Henry Skorna

**Affiliations:** 1 Collaborative Research Center 1266, Kiel University, Kiel, Germany; 2 Institute of Pre- and Protohistoric Archaeology, Kiel University, Kiel, Germany; 3 Department of Historical Studies, University of Gothenburg, Gothenburg, Sweden; 4 Cluster of Excellence ROOTS, Kiel University, Kiel, Germany; 5 Landesamt für Denkmalpflege und Archäologie Halle (Saale), Halle, Germany; University of California Santa Cruz, UNITED STATES

## Abstract

The production, distribution and use of copper objects and the development of metallurgical skills in Neolithic Northern Central Europe and Southern Scandinavia are linked to early centres of copper metallurgy of South East Central Europe and Southeast Europe. A total of 45 Neolithic copper objects, until now the largest sample of Early Neolithic objects from the Northern Central European Plain and Southern Scandinavia, were selected for new lead isotope analyses. They aided in the identification of the origin of the copper: These new analyses indicate that the copper ore deposits in Southeastern Europe, especially from the Serbian mining areas, were used for the Early Neolithic northern artefacts (ca. 4100–3300 BC). The most likely sources of copper for the few Middle Neolithic artefacts (ca. 3300–2800 BC) seem to be from the Slovak Ore Mountains, the Serbian mining areas and the Eastern Alps, whereas deposits of the Slovak Ore Mountains and the Alpine region were used for the Late Neolithic and the Early Bronze Age (ca. 2300–1700 BC) artefacts. For the artefacts dated after 2000 BC, the Great Orme mine in Wales also appears to have been the source of copper for the analysed metals. The use of copper from different regions of Europe probably reflects changing social and cultural connectivities on a European scale and the changing chronology of copper exploitation.

## 1. Introduction

Early copper artefacts are considered to have a high cultural and historical significance in European prehistory. In addition to purely technological advances, subsequent transformations in economic and social developments have been postulated. These include the development of forms of work division and the necessity of wide-scale exchange networks, but also the strengthening of social inequality in Neolithic, Chalcolithic and Bronze Age societies [1–10]. The question if the appearance of copper artefacts can be equated with the introduction of metallurgical processes or “only” the exchange of implements is of vital importance in order to understand social transformation processes within the receiving societies. Recent studies highlighted the socio-economic impact of metallurgy by characterising the Bronze Age as the first pre-global or even global phase of history [11, 12]. However, the significance of analyses of the origin of copper artefacts for the reconstruction of long-distance connections is shown by the study of the lead isotope ratios of the copper axe of the Alpine Iceman, which concluded that the axe material was extracted from Southern Tuscan ores and not from Alpine ores in the Alpine region of the site [13].

In Europe, the oldest evidence for metallurgy is known from the Southeastern Balkans with the first mining of copper ores around 6200 BC [14], earliest evidence for smelting around 5000 BC [14] and a metallurgical boom around the mid-5^th^ millennium BC [15–17]. Around 4300 BC, copper metallurgy reached the Carpathian Basin and East Central Europe; evidence of the local production of copper artefacts is known from South Central European contexts since around 3800 BC [18]. In this respect, a socio-chronological terminology of archaeological periods was postulated for Europe: Not only the Neolithic (with copper imports, but no copper metallurgy) but also the Chalcolithic (with copper metallurgy, but no significant social changes of the society) and the Bronze Age (with copper metallurgy propelling social changes) were defined as socially determined periods [3]. From a technological perspective, a “preliminary stage” (the usage of coloured stones including copper minerals), an “initial phase” (first metallurgy using native copper), an “innovation phase” (early metallurgy consisting of smelting oxide ores in simple vessels) and a “consolidation phase” (developed metallurgy) were differentiated [3, 19].

For the Northern European Plain and Southern Scandinavia, the importance of copper artefacts and their origins have been intensively discussed for decades [3, 20–23]. In contrast to Southeastern Europe, where first copper objects made of malachite and azurite appear around the late 7^th^ and the early 6^th^ millennium and the mining, smelting and casting of copper are attested around 5000 BC [6, 20, 24], copper items did not reach Northern Central Europe/Southern Scandinavia until the end of the 5^th^ millennium BC. Here, it is of particular importance that on the Northern European Plain and in Southern Scandinavia, copper deposits had not been exploited during the Neolithic or Bronze Age [25] in the area, so that any raw material would have to have been imported [5, 26]. This also applies to the island of Helgoland, whose flint was transported to the mainland in the Neolithic [27], but there is no evidence that the island’s copper resources were exploited in the Neolithic [18].

**Fig 1:**
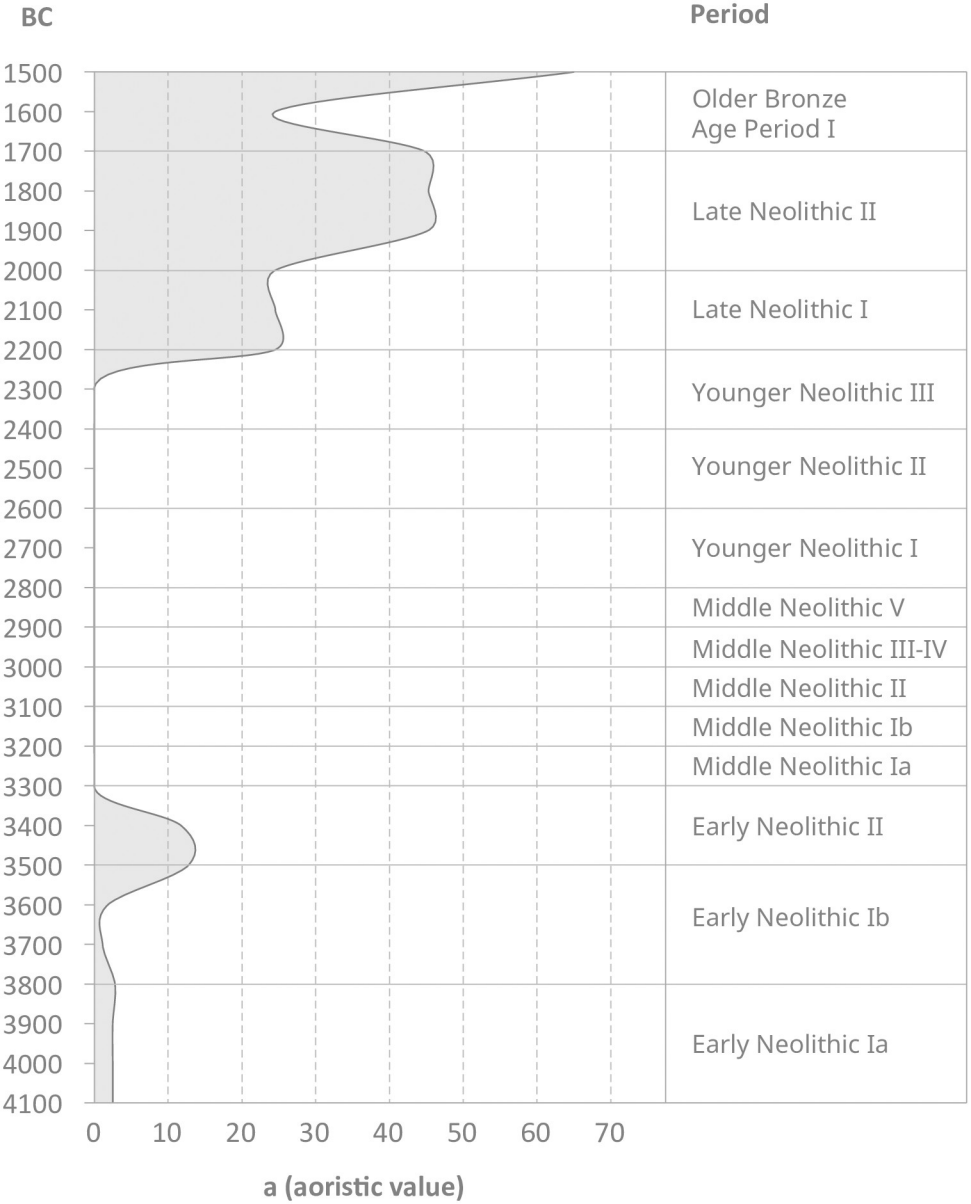
Relative frequency (number of objects per century) of Neolithic copper objects in Northern Germany and Southern Scandinavia (database: [18, 29, 30]). The duration of the archaeological periods in Southern Scandinavia and North Central Europe are marked.

On the North Central European Plain and in Southern Scandinavia, an increase in copper artefacts is evident from 4100/4000 BC onward with the advancing process of neolithisation (Fig 1), especially with a peak from 3500–3300 BC [18]. In Northern Central Europe and Southern Scandinavia, metallurgical skills, such as cold forging (including the production of local types), and pyrotechnical processes, such as copper smelting, can be reconstructed for this phase [20]. The earliest crucible is known from the late Early Neolithic around 3500 BC [28].

From about 3300 BC onwards, a sharp decline in imported material and a discontinuation of local processing can be observed [18]. This lasted for almost 1000 years, until a local metal processing economy evolved again around ca. 2350 BC. New exchange routes emerged, which became increasingly important from 2000 BC on and into the Bronze Age. These linked the economies of the north primarily with the Únětice groups, but also with the British Isles. Thus, in a recent lead isotope study especially for the Scandinavian Late Neolithic and the Bronze Age, the incoming metal flow both from the British Isles as well as from Alpine and Slovak copper ores was proven [4, 22, 26]. Concentrations of Baltic amber in Central Europe suggest a complex network of import and export routes based on a system of intermediaries [4, 11, 31–33], in which metal played only one part.

Analyses of the metal composition of Bronze Age artefacts from Northern Europe were initially carried out mainly within the SAM metal analysis project [34]. Continued later on, the FSDM project was not able to trace the origins of the raw material used during the Neolithic, but it was suggested on very weak evidence that the copper mainly came from deposits in Southeastern Europe and it was identified as a type of arsenical copper–the so-called “Mondsee copper”–located in the Eastern Alps [4, 18, 35–38]. Then in 1982, Gale and Stos-Gale published a paper suggesting that copper alloys can be linked to the source of copper by using lead isotope analyses [39].

While the trace elemental compositions of ore deposits can be very diverse in one region of mineralisation, and quite similar across a range of localities, and the trace element pattern of ore and metal smelted from it can also be significantly different due to the extraction process [40], for lead isotope analyses, such problems do not exist. It has been proven that the lead isotope compositions during the metallurgical processes ([3], 59, [35, 39]) of mixing ores or scrap metal from different sources alters the isotopic “fingerprint” [41].

After many years of scientific research and controversial disputes, comparative lead isotope and chemical analyses of ancient metals and ores from known ore deposits are now accepted as the best method to determine the origin of copper, lead and silver from archaeological contexts [42].

The method of interpretation of analytical data relies on comparisons of geochemistry and lead isotope ratios of ancient artefacts with analytical data for minerals from ore deposits. The comparisons of data in this project are based on calculations with the Euclidean distances of three independent isotopic ratios, measured for a sample of the metal under study, with all the ores in the database of lead isotope ratios of samples of ores from clearly defined mineralisations. This process enables a selection of ore deposits that geochemically could be the source of the tested metal, after which deposits can be eliminated that were not exploited in a given epoch [22]. The current database of isotopic ratios for ores related to the archaeology of Europe and the Middle East contains more than 9,000 sets. There are only two ‘open access’ lists of isotopic data applicable to provenance studies of ancient metals: one, OXALID [43], can be found on the website of the Isotrace Laboratory of the University of Oxfordhttp://oxalid.arch.ox.ac.uk/, the other, IBERLID [44], has been assembled in the Geochronology and Isotope Geochemistry Facility of the Universidad del Pais Vasco and presents all data published for the Iberian ore deposits and ancient metals.

In principle, the metal of an artefact can be considered completely consistent as to the origin of a particular ore deposit if its three independent lead isotope ratios are identical to the ore samples from that deposit and its chemical composition is consistent with the mineralogy of this particular ore. In addition, the periods of mining activities in the selected deposit should also be taken into account. The isotopic ratios of artefacts and ores are visually further compared on two-dimensional plots of corresponding ratios (for example: ^207^Pb/^204^Pb versus ^206^Pb/^204^Pb and ^208^Pb/^204^Pb versus ^206^Pb/^204^Pb).

Not all ore deposits have different ranges of lead isotope ratios, so it is not uncommon for the data for ores from different regions to be similar. In this case, two main conditions should be considered: the geochemistry of the ores in each of the deposits and the likelihood of their exploitation during the period under consideration. The elemental compositions of metals reflect the type of mineral ore used for smelting, and typical impurities are often characteristic of specific deposits. The most important impurities associated with mineral types are nickel (Ni), antimony (Sb), arsenic (As) and silver (Ag). These elements, sometimes together with zinc (Zn) and gold (Au), are most often a geochemical indicator of ore deposits. In addition, trace element signatures help to distinguish metal groups that do not only reflect different types of ores but are also important for comparisons with previously analysed artefacts from the same period. It should be stressed, however, that identical characteristics of lead isotopes do not guarantee that their elemental compositions will also form a group (or vice versa).

The major study by Klassen and Stürup on the so-called Neolithic “Riesebusch” copper [35] marks a water-shed in the application of lead-isotopes in the analysis of early metallurgy in Northern Europe. With 38 analyses, it included a large part of the then-known relevant objects. Unfortunately, a re-analysis of some of the objects by Nørgaard *et al*. [26] showed that these early analyses have to be regarded as unreliable. The papers by Nørgaard *et al*. [4, 26] focussed on the Late Neolithic and the Early Bronze Age. Also included were eleven samples from Early Neolithic, Middle Neolithic and Younger Neolithic periods (nine are dated between 3800–2850 BC, two are dated between 2850–2300 BC; of these, nine are re-analyses of the samples already measured by Klassen and Stürup [35]) and two are from the early Late Neolithic (LNI, 2350–1950 BC).

Thus, details of the origin of the raw material especially for Early Neolithic copper artefacts from the Northern Central European Plain and partly Southern Scandinavia remain cloudy and need to be reassessed. The same holds true for the extent of the involvement of the north in the reconstructed copper-based, pan-European network of raw material exchanges.

In order to determine the provenance of the raw material, in this study additional lead isotope (MC ICP-MS) and X-ray fluorescence (EDXRF) analyses of 45 copper objects of the 4^th^ and 3^rd^ millennia BC were carried out and compared with the geochemical characteristics of prehistoric copper mining sites. Thus, if we reject the early analysis by Klassen and Stürup [35], our sample effectively more than doubles the number analysed so far for the Early and Middle Neolithic of Northern Europe. Results of this determination of the provenance of copper and their discussion with respect to the socio-cultural responses of Neolithic societies on the Central European Plain and in Southern Scandinavia provide the focus of this paper.

## 2. Materials and methods

For our study, 45 Neolithic copper objects from northern Central Europe and Southern Scandinavia were selected from the database of the SAM metal analysis project (S1 Table). Objects with low lead contents and insufficient results, samples that had already been measured by Nørgaard *et al*. [26], and not available material had to be excluded. However, we re-analysed 15 samples that were already measured by Klassen and Stürup [35], as Nørgaard *et al*. [26] have shown that these early analyses are unreliable.

The study area is limited to the south by the Central European Lower Mountain Range, to the west by the Ems River, to the east by the Oder River, and to the north by the Skåne Peninsula, forming part of the geological Baltic Shield to the northwest. Of the analysed objects, 26 are from Germany, 12 from Denmark, and 7 from Sweden.

The analysed objects include 33 flat axes of various Southeast European and local types. In addition, there are three flanged axes, one axe of Jászladány type, one chisel, three Zabitz-type double axes, three spirals, and one spiral roll (cf. S1 Table). Of these, the majority (35 objects) are of single find context. These include nearly all of the flat axes, as well as two Zabitz axes, one Jászladány-type axe and one chisel. Only nine of the analysed objects belong to deposits consisting of more than one artefact. One flat axe and three spirals belong to the deposit of Ratekau-Riesebusch ([18, 45], 88–90]). One further flat axe was sampled from the Neuenkirchen deposit [46]. One of two double axes of the Zabitz-type deposited together in Grastrup-Hölsen was also sampled [47, 48]. A single spiral from the megalithic grave of Emmeln 2 belongs to an inventory of at least 384 vessels, as well as additional tubes of copper and amber beads [49, 50]. The dating of the finds was based on typo-chronological comparisons and find associations [18, 29, 49–54].

The analyses were performed using lead isotope (MC-ICP-MS) and X-ray fluorescence analysis (EDXRF) methods at the Curt-Engelhorn-Zentrum Archäometrie gGmbH in Mannheim. The interpretation of lead isotope analyses was based on comparisons of the lead isotope ratios for each artefact with a database of over 10,000 lead isotope ratios for the copper and lead ores from Europe and the Near and Middle East which used TestEuclid calculations [22]. The implemented database was assembled by one of the authors (ZS-G) and includes all data on the open data bases OXALID [43] and IBERLID [44] as well as other relevant published data and databases shared with colleagues involved with archaeological lead isotope research (G. Artioli, F. Cattin and others).

## 3. Results—Scientific analyses

### 3.1. General comments on the chemistry of the analysed artefacts

The chemical compositions of the artefacts analysed for this project allow us to state that they are not made of alloyed metals. Rather, the copper impurities are characteristic of the respective ores from which they were extracted. Out of the 40 artefacts, which were analysed for their chemical composition, 39 contain lead in quantities below 0.1%. Only one double axe from Börssum (S1 Table, ID 10) consists of about 0.2% lead (cf. S1 Table). Therefore, the laboratory specifies an analytical precision between 10 and 20%. Only seven samples contain antimony (Sb) in excess of 0.5%. The same artefacts contain silver (Ag) in quantities between 0.5–1%, arsenic (As) between 0.2–0.8%, nickel (Ni) between 0.2–2%, and tin (Sn) between <0.1–0.24%. These artefacts were obviously made of copper smelted from minerals containing mainly Sb and Ag as impurities. Such ores are usually referred to as ‘*Fahlerz*’ in German. The most prominent mines exploited in the 4^th^-3^rd^ millennia BC, which contained these ores, are located in the North Tyrol region of Austria. Indeed, five of our artefacts display lead isotope compositions consistent with copper originating from the North Tyrol region, but a flat axe from Ulvemosehusene (S1 Table, ID 30) containing 0.5% Ag and 1% Sb and a flanged axe from Harpe in Saxony-Anhalt (S1 Table, ID 44) containing 0.96% Ag and 0.47% Sb show lead isotope compositions of copper minerals which are much older than the ones from Tyrol. Some 30 artefacts contain As in quantities above 0.1%: the highest value here is 1.7%, the average 0.5%. In nearly all cases, all the other analysed trace elements of our copper samples are present in quantities below 0.1%.

### 3.2. The origin of the raw material

In addition to an object-based, typo-chronological categorisation, the samples were also differentiated into four chronological groups for the evaluation of raw material provenance. These encompass four chronological periods: ca. 4000–3500 BC (Group 1), ca. 3500-3000/2800 BC (Group 2), ca. 2800/2300-2000 BC (Group 3) and ca. 2000–1700 BC (Group 4). The first group is associated with the local Earliest Neolithic, the so-called early “Trichterbecherkultur” (TRB, funnel beaker “culture”). The second group encompasses the Middle Neolithic, including the middle and late TRB, the third group the Younger and the early Late Neolithic, including Single Grave societies and Dagger groups, and the forth group the later Late Neolithic and the Older Bronze Age, including younger Dagger groups and the earliest Nordic Bronze groups. A discussion of the typo-chronological dating of individual objects and their provenance is provided in Section 4.

#### Group 1, ca. 4000–3500 BC

**Fig 2:**
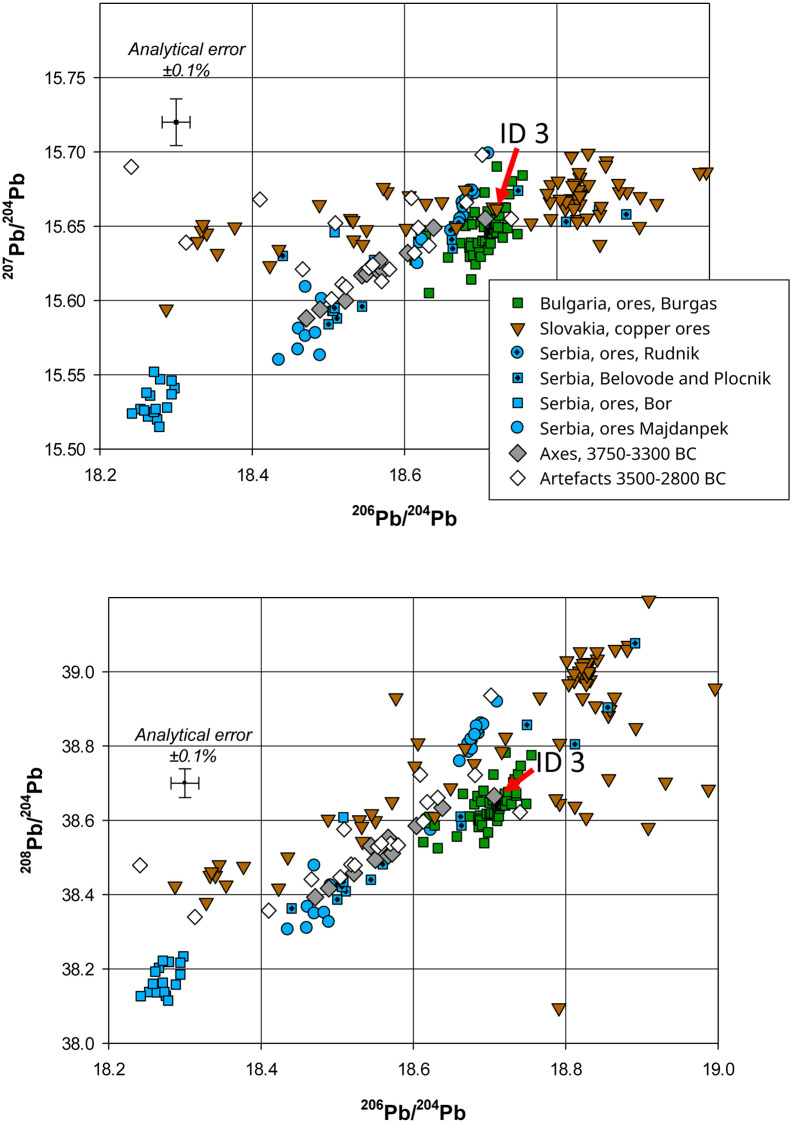
Comparison of the lead isotope ratios for non-radiogenic ^204^Pb for the artefacts in Groups 1 and 2 with the data for copper ores from the Chalcolithic copper mines in the Balkans and in the Slovak Ore Mountains. The finds of ores from the Eneolithic sites of Plocnik and Belovode are also included (all data for Serbia is from Radivojević *et al*. 2021 [58]; data for Slovakia is from Schreiner 2007 [59] and for Bulgaria from Stos-Gale *et al*. 1998 [60]. The axes dated to the 4^th^ millennium BC are isotopically and chemically fully consistent with the copper ores from Serbia and Bulgaria.

**Fig 3:**
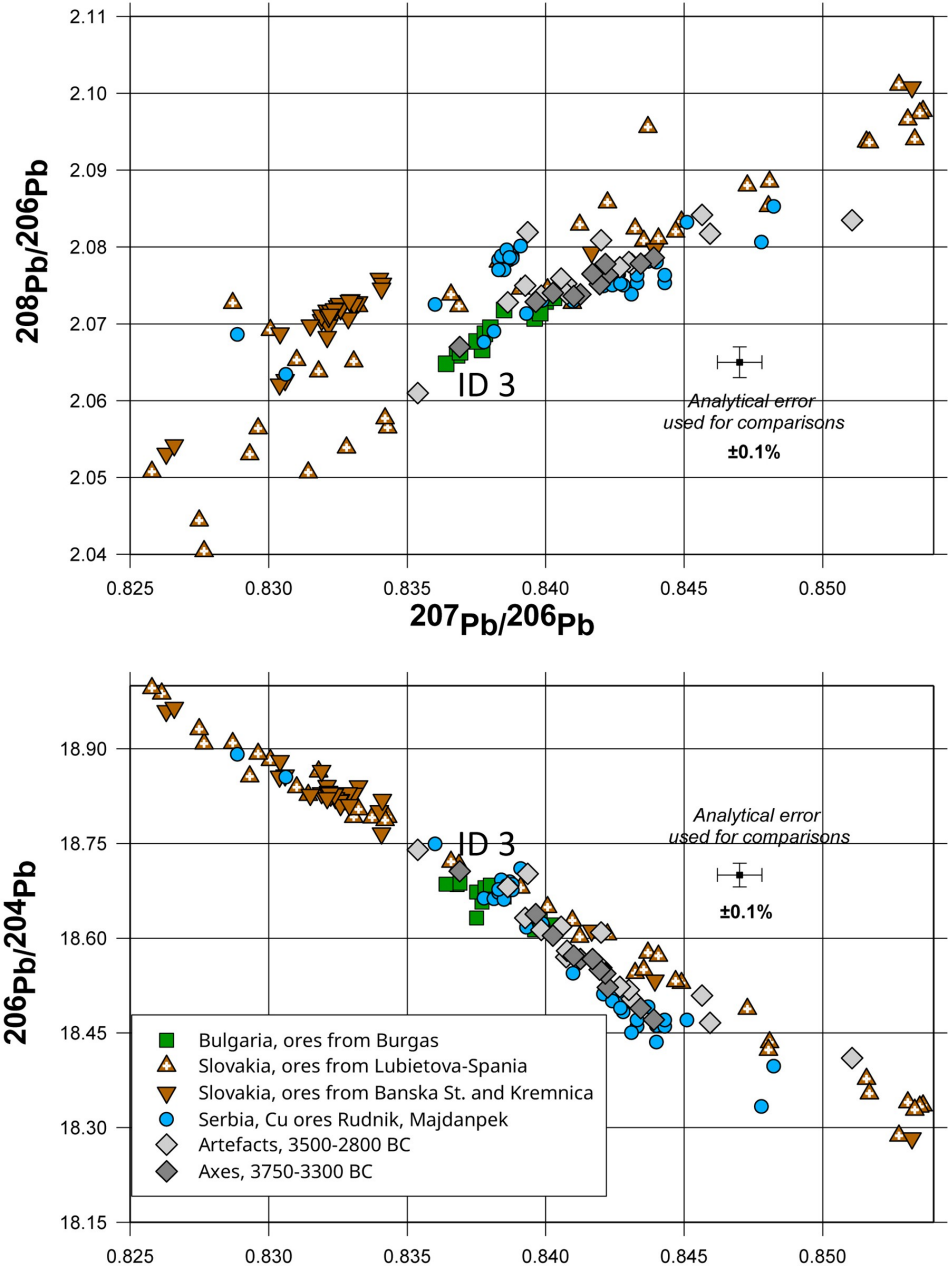
The plot of the data for the same artefacts with respect to the radiogenic lead isotope ratios confirms the consistency with the origin of the 4^th^ millennium axes from Group 1 with the ores from Serbia and, in the case of the axe from Pantelitz (S1 Table, ID 3), with the ores from the mines near Burgas in Bulgaria. The metals from Group 2 display a much wider range of lead isotope ratios. In this plot, the range of ^207^Pb/^206^Pb represents the artefacts with ratios consistent with the ores from the Slovak Ore Mountains and Serbia.

**Fig 4:**
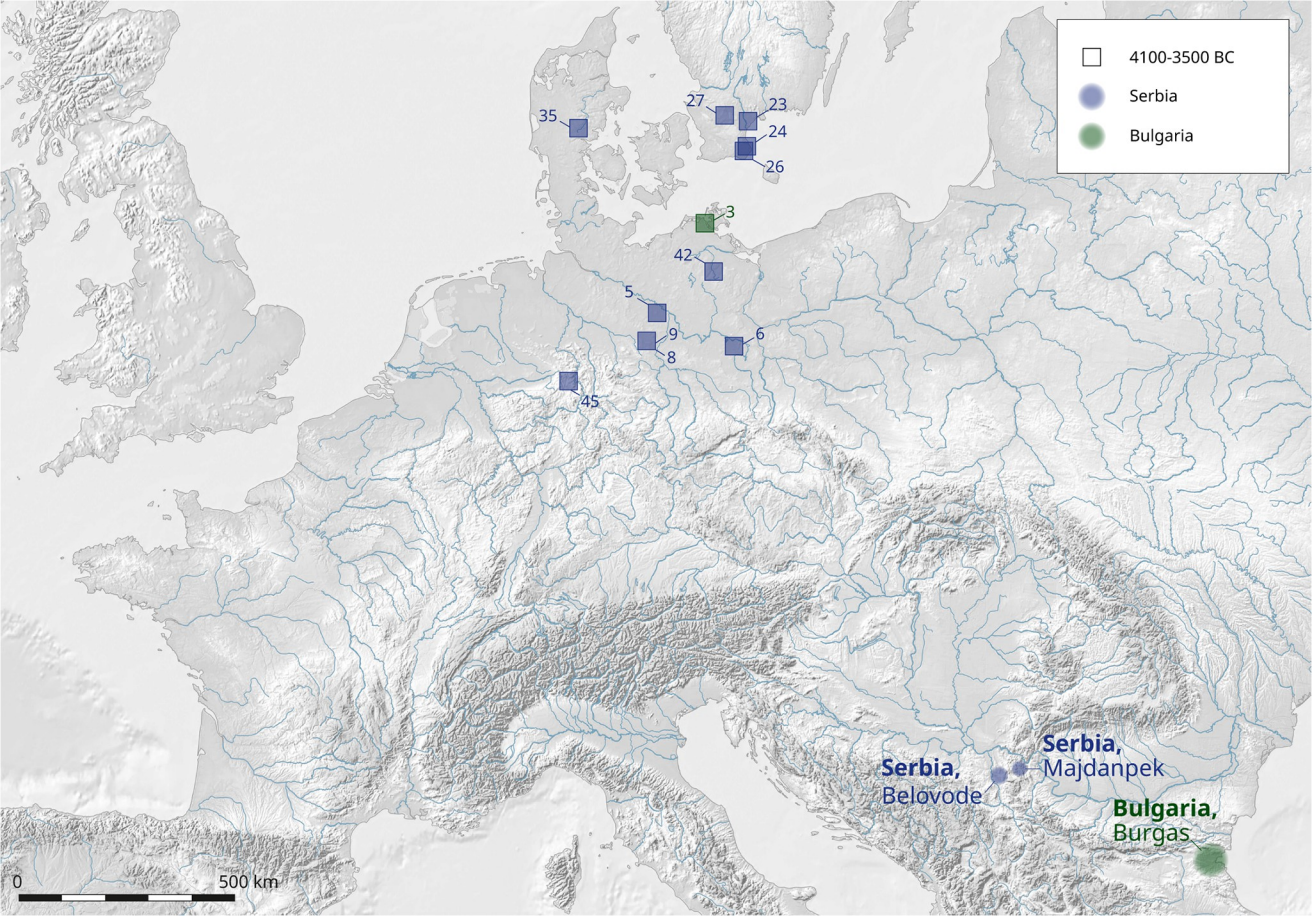
Locations of the sites of the sampled objects of Group 1 (ca. 4100–3500 BC) in the study area and the regions of origin of the copper (cf. S1 Table) based on the analyses of this study [IMAGINE TIFF support, copyright 1991–1999 by ERDAS, Inc. All Rights Reserved, @(#)$RCSfile: etif.c $ $Revision: 1.9.1.3 $ $Date: 2002/07/29 15:39:06EDT $] (C. Reckweg, CAU Kiel).

Eleven of the analysed artefacts belonging to this chronological group were made from very pure copper containing small amounts of arsenic (0.01–2.5%). Moreover, this group also contains an axe from Viby (S1 Table, ID 23) for which we only have lead isotope data, but no chemical data. Calculations of the Euclidean distances between the lead isotope ratios of each of these artefacts and the data for over 10,000 ore samples from the ore deposits in Europe and the Near and Middle East resulted in the conclusion that apart from a single example (S1 Table, ID 3, an axe from Pantelitz), the copper for all of these axes most likely originated from the copper deposits in the Majdanpek region of Serbia [55, 56] (Figs 2 and 3). The axe from Pantelitz is fully consistent with the lead isotope ratios of the ores from the Burgas region of Bulgaria [57] (Fig 4).

#### Group 2, ca. 3500-3000/2800 BC

**Fig 5:**
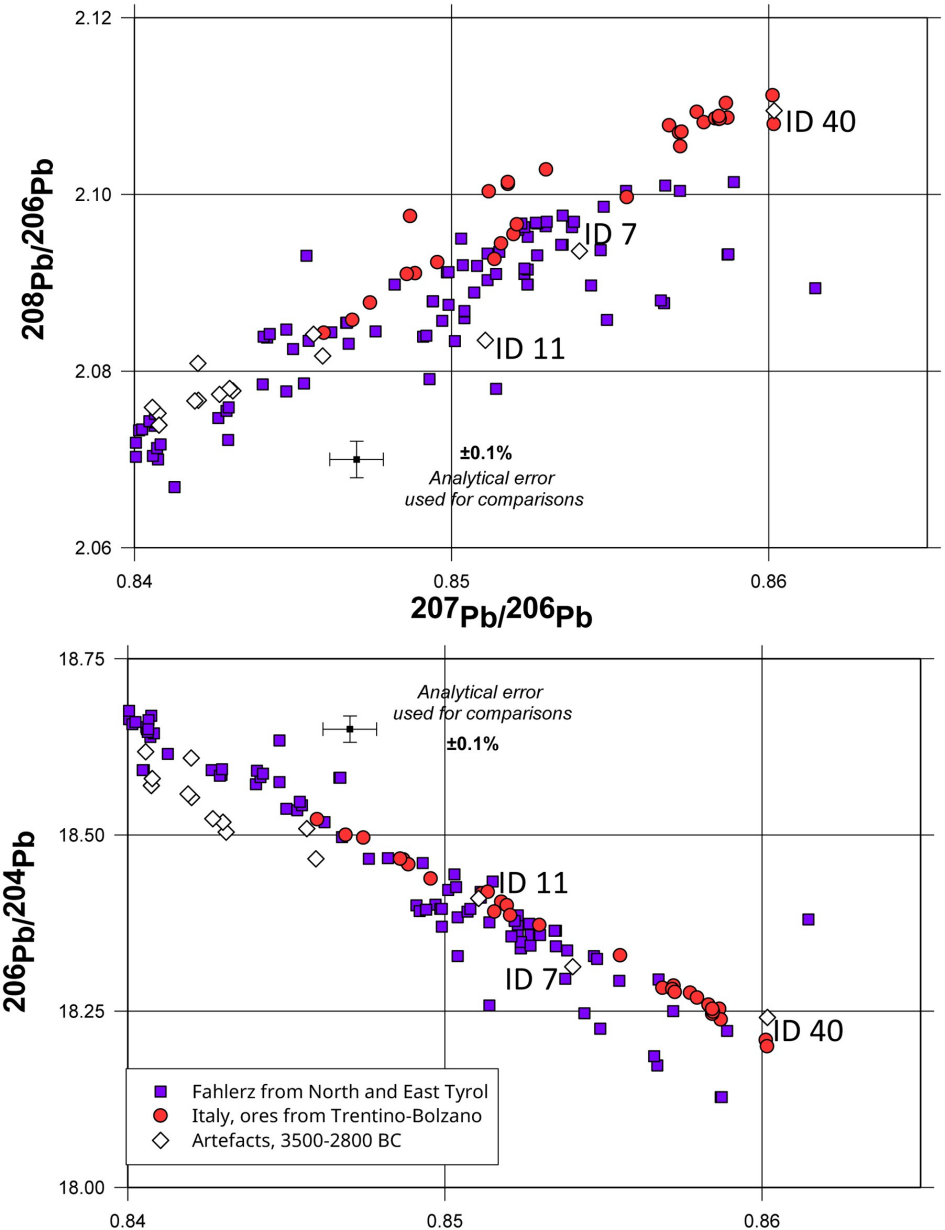
Comparison of the lead isotope ratios of the artefacts from Group 2 with the data for the copper ores from North and East Tyrol [66] and the Italian Eastern Alps (Trentino-Bolzano region) [67]. The range of the ^207^Pb/^206^Pb represents the range of the lead isotope compositions of ores from these localities.

The chemical compositions of 17 artefacts in this group indicate the use of very pure copper, with small quantities of arsenic (0.01–1.4%, with an average of about 0.5%). Exceptions are provided by two flat axes, one from Tremmen (S1 Table, ID 7) and one from Herbede (S1 Table, ID 11), which contain 0.97% and 0.62% silver respectively, as well as 0.7% and 0.2% antimony. The two elements indicate the use of *Fahlerz* ores. These two axes display a somewhat different lead isotope ratio which would be fully consistent with ores from Wales (Parys Mountain and the Great Orme [61, 62], however, it seems unlikely that they originate from these sources due to the high content of silver and antimony. Alternative Bronze Age mines, which produced *Fahlerz* with related lead isotope ratios, can be found in Le Valais in Switzerland [63–65] and the ore deposits of the Austrian Alps in North Tyrol [66]. In view of the established chronology of copper smelting in the latter two locations, it seems most probable that the copper for our sampled axes originated in the Inn Valley of North Tyrol (Fig 5). The axe from Hårby (S1 Table, ID 40) displays lead isotope and chemical characteristics consistent with the ores of the Eastern Alps in Italy’s Trentino-Bolzano region [65].

**Fig 6:**
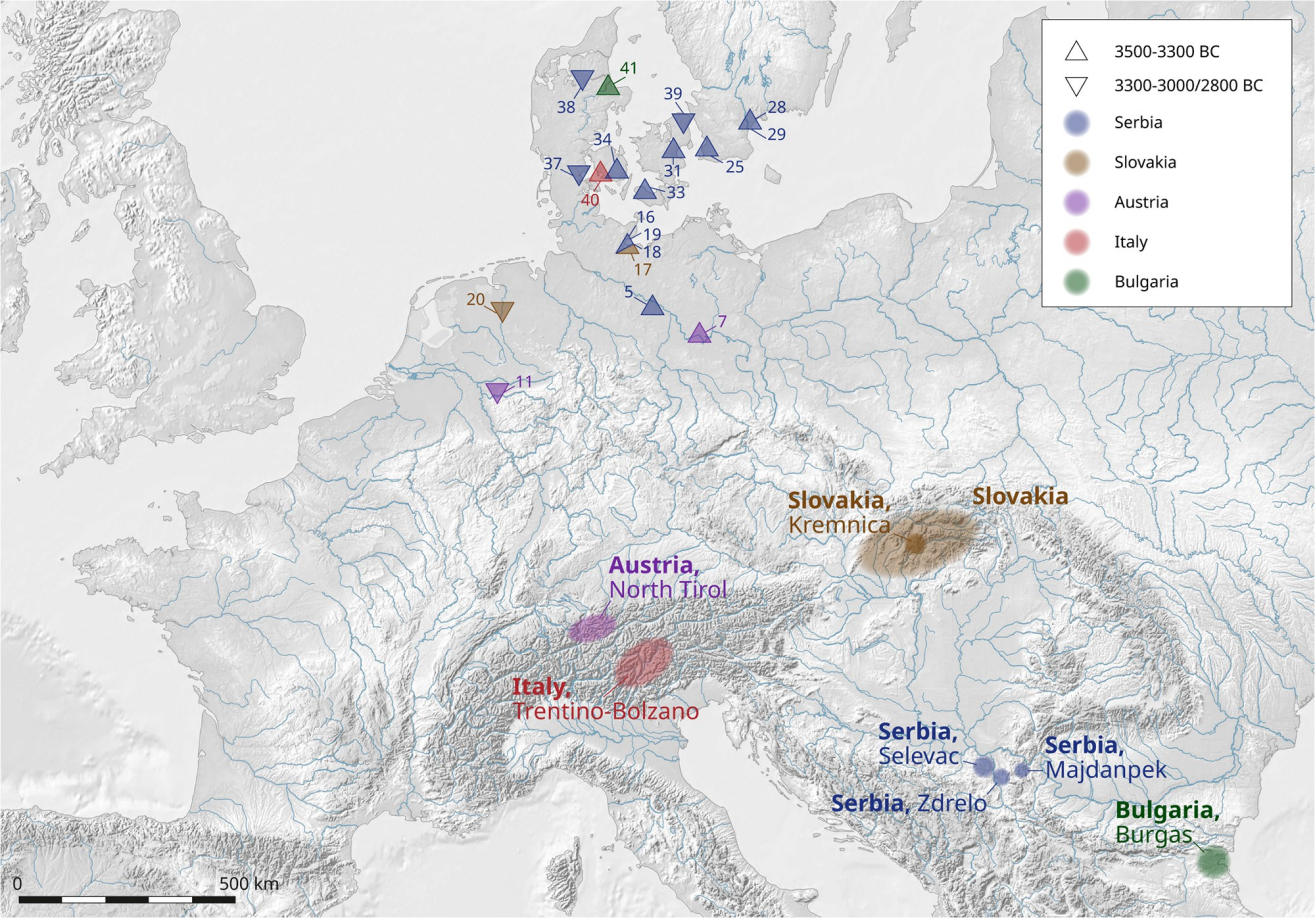
The sites of the sampled objects of Group 2 (ca. 3500–3300 BC and 3300-3000/2800 BC) in the study area and the regions of origin of the copper (cf. S1 Table) based on the analyses of this study [IMAGINE TIFF support, copyright 1991–1999 by ERDAS, Inc. All Rights Reserved, @(#)$RCSfile: etif.c $ $Revision: 1.9.1.3 $ $Date: 2002/07/29 15:39:06EDT $] (C. Reckweg, CAU Kiel).

Two spirals from Ratekau-Riesebusch (S1 Table, ID 17 and 19) are consistent in their compositions with ores from the Kremnica region in the Ore Mountains of Slovakia, while the flat axe (S1 Table, ID 16) and another spiral (S1 Table, ID 18) possess compositions with ores from Majdanpek. The axe from Møllebakken (S1 Table, ID 41) has lead isotope ratios conforming to the ores from the region of Varly Briag in Bulgaria (there is no chemical analysis). The remaining 13 artefacts are isotopically consistent with the ores from Serbian deposits in the region of Majdanpek (Fig 6).

#### Group 3, ca. 2800/2300-2000 BC

**Fig 7:**
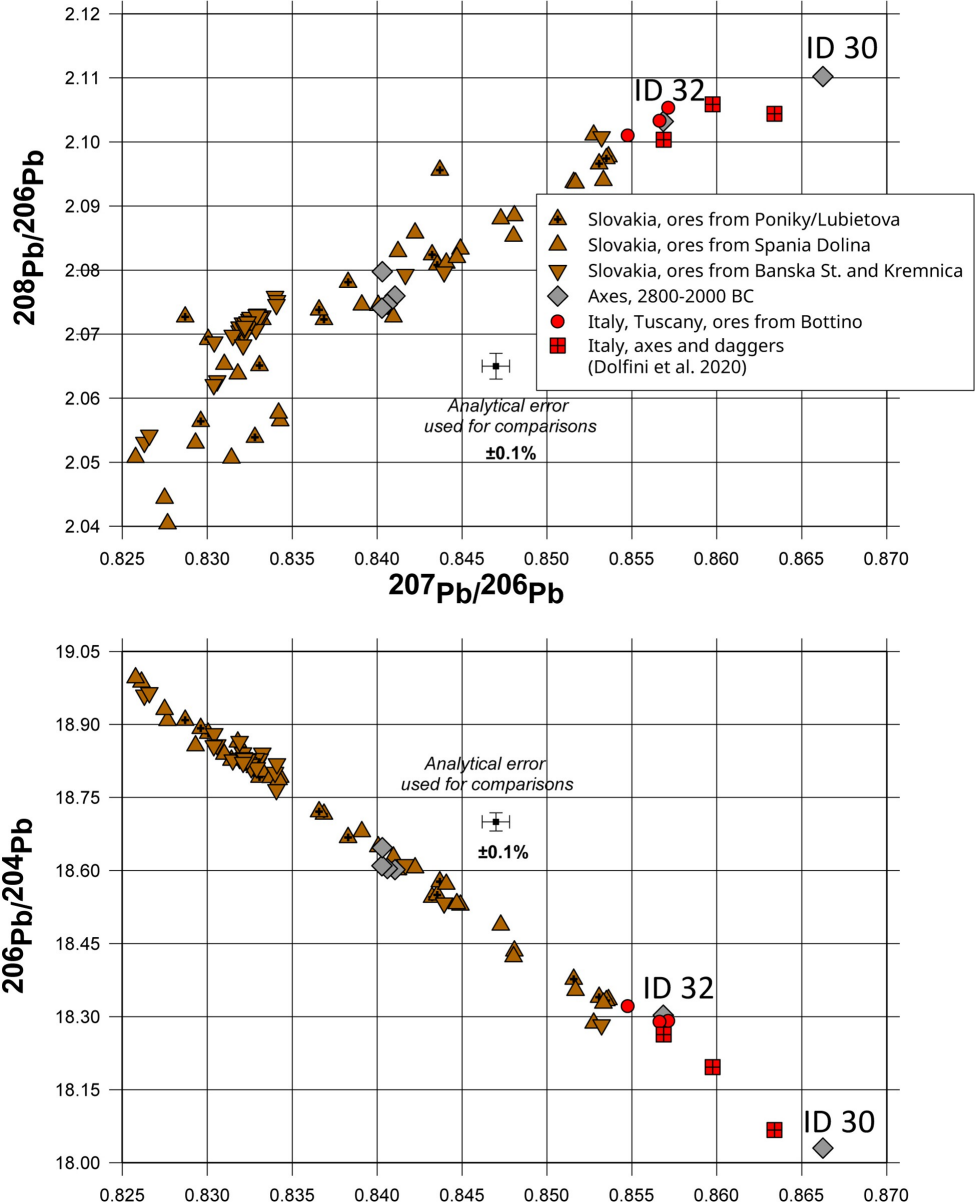
Comparison of the lead isotope ratios for the axes from Group 3 with the data for the ores from the Slovak Ore Mountains [59]. The lead isotope ratios for the two flat axes found in Denmark are similar to contemporaneous copper artefacts from Northern Italy, indicating that they were most likely made of copper from the Western Alps and Tuscany [68].

This group consists of only six axes, of which three are double axes and three flat types. The three double axes, which were found in three different locations (Börssum, S1 Table, ID 10; Gastrup-Hölsen, S1 Table, ID 21 and Ketzin, S1 Table, ID 22) display nearly identical chemical compositions and lead isotope ratios, which are fully consistent with the ores from the Ore Mountains in the Slovakian Banska Stiavnica-Kremnica region (Fig 7). The chemical signature could indicate that the axes were cast from the same batch of metal: the differences in the content of arsenic and lead are small enough to be attributed to the inhomogeneity of the metal used, or to segregation and evaporation during casting. The flat axe from Vietznitz (S1 Table, ID 1) has a lead isotope and chemical composition, which is also fully consistent with the ores from Spania Dolina or Kremnica in the Slovak Ore Mountains.

The other two flat axes, which were found in Denmark (S1 Table, ID 30 and 32), have lead isotope ratios indicating an origin of their copper from geologically much older ores, similar in age to those of the Italian Alps, Sardinia, or Southern Spain. However, one of them (S1 Table, ID 30) contains significant impurities of antimony and silver (1% and 0.57% respectively), indicating that the copper was smelted from tetrahedrite minerals (Fahlore), which were not smelted in the Eastern Alps of Italy or on Sardinia during the Bronze Age [69–71].

The axe from Flinterupgård (S1 Table, ID 32) displays a lead isotope composition identical to the ores from Catalonia-Molar, but there is no evidence of the exploitation of these copper deposits during the Bronze Age, when these mines were mainly mined for lead [72]. In contrast, the composition and somewhat higher silver content of the axe is consistent with the ores from the copper mine of Bottino in Tuscany, where copper was mined during the 3^rd^ millennium BC [73].

**Fig 8:**
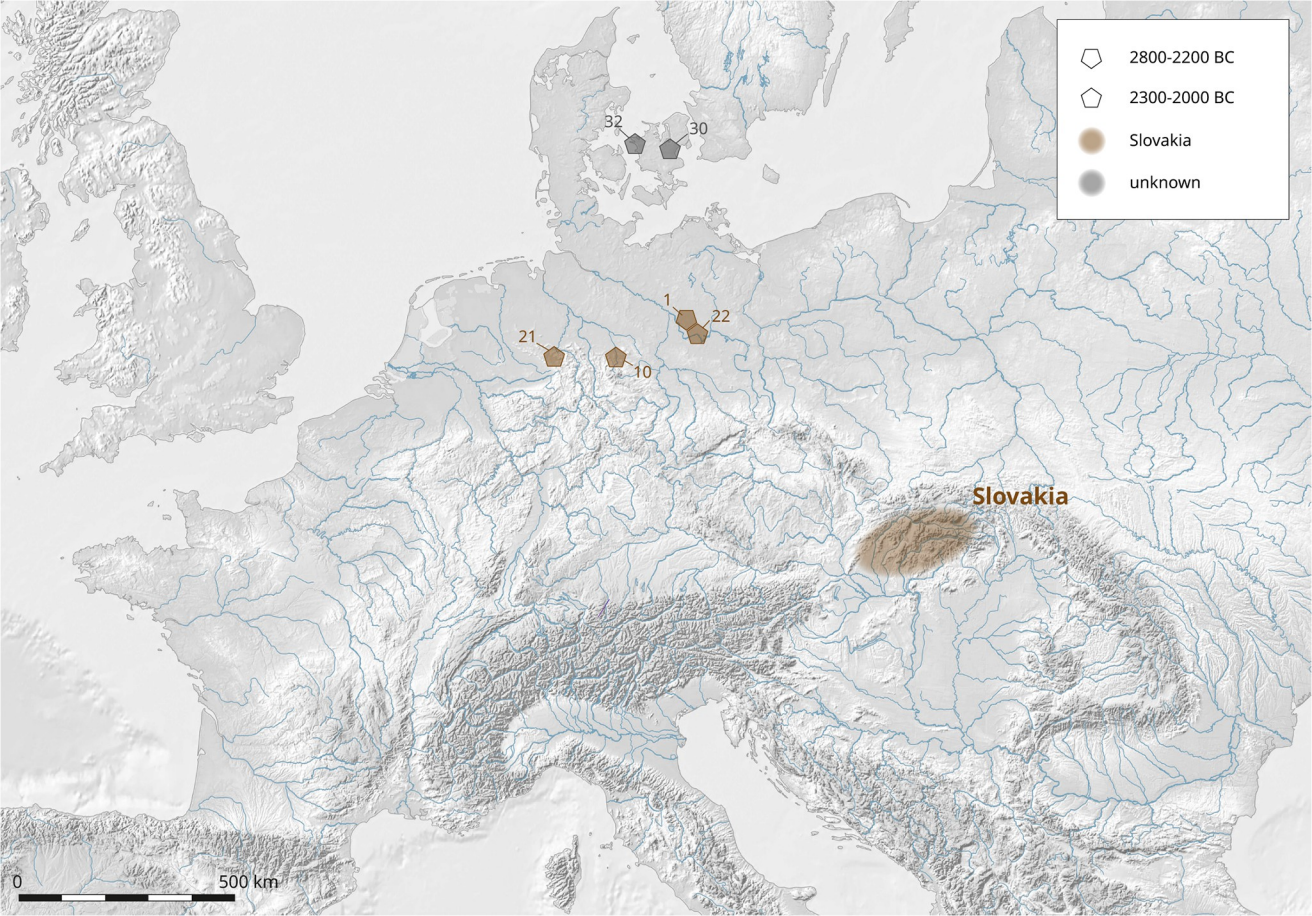
The sites of the sampled objects of Group 3 (ca. 2800/2300-2000 BC) in the study area and the regions of origin of the copper (cf. S1 Table) based on the analyses of this study [IMAGINE TIFF support, copyright 1991–1999 by ERDAS, Inc. All Rights Reserved, @(#)$RCSfile: etif.c $ $Revision: 1.9.1.3 $ $Date: 2002/07/29 15:39:06EDT $] (C. Reckweg, CAU Kiel).

**Fig 9:**
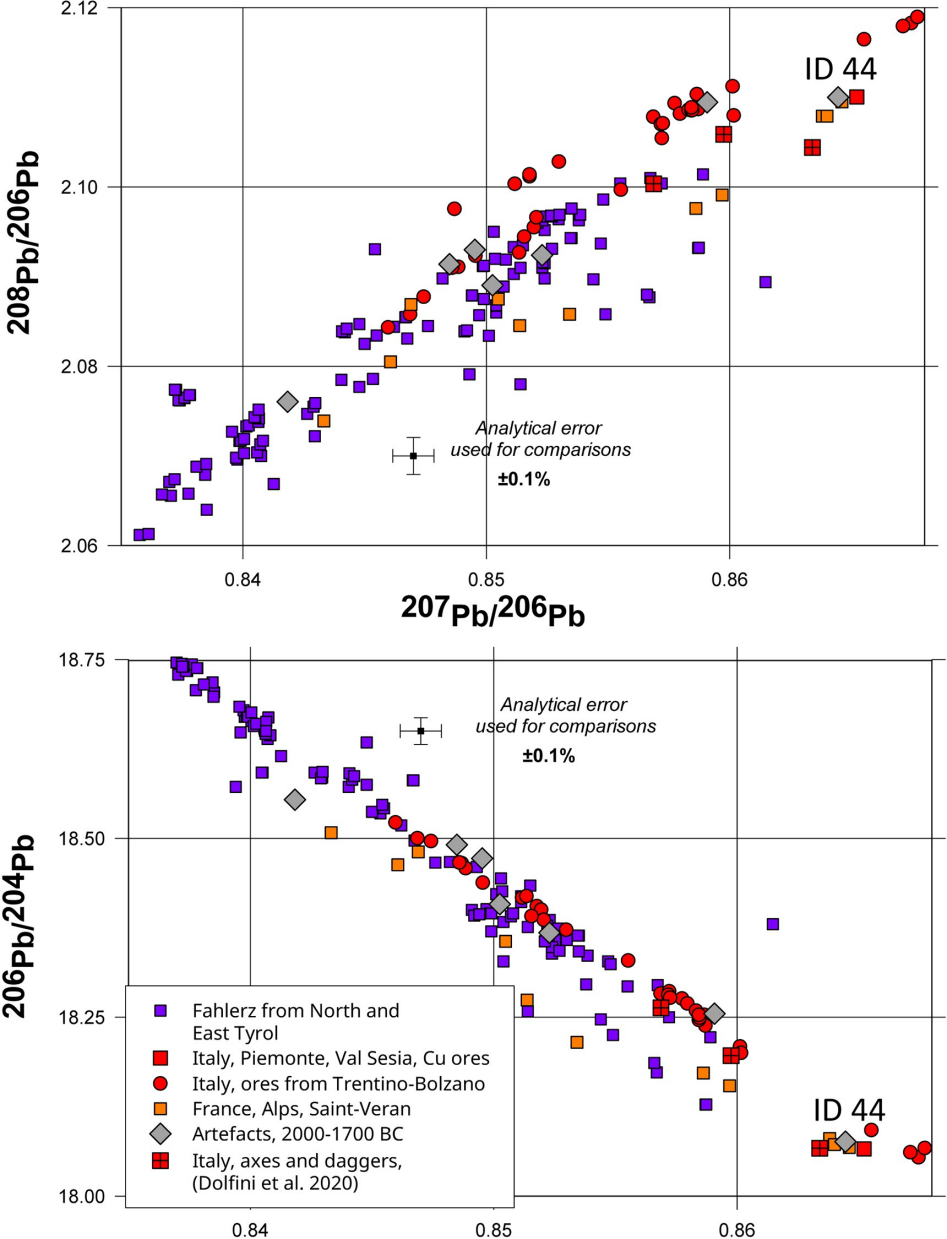
Comparison of the seven artefacts from Group 4 with the data for the ores from North Tyrol [66] and the Italian Alps (Trentino-Bolzano) [67] which best correspond to their lead isotope ratios. Only a few ore samples from the mines of Saint Veran [74] are isotopically identical with the artefacts found in Northern Europe, but they do not match the composition of copper of these axes chemically.

In comparing the analytical data for the 3^rd^ millennium axes and daggers found in Northern Italy published by Dolfini *et al*. [73] with the metal compositions of the two axes from Denmark, it seems quite possible that both groups of artefacts were made from copper that was smelted in the Western Alps of Northern Italy. In Fig 9, the lead isotope data for a dagger and two axes (Que-Pg, PGr-Ax, Gar-Axg) are compared with the data for our two axes from Denmark; this comparison indicates a certain likelihood of an origin of all of these artefacts from the same source of copper ores. Significantly, the three artefacts from Italy display not only lead isotope ratios, but also chemical compositions which are similar to the two axes found in Denmark ([73], Table 5) (Fig 8).

#### Group 4 (ca. 2000–1700 BC)

This group of nine copper artefacts shows a greater variety in the origin of their material. One flanged axe (S1 Table, ID 12), one flat axe (S1 Table, ID 4) and a chisel (S1 Table, ID 36) have lead isotope and chemical compositions which are a good match for the ores from North Tyrol [66]. There is no chemical analysis for the flat axe from Wustermark (S1 Table, ID 13), but it is isotopically consistent with the ores from the North Tyrol region. The flanged axe found in Leppin, Saxony-Anhalt (S1 Table, ID 43) appears to be consistent with the *Fahlerz* ores from North Tyrol. The flat axe from Tarmov (S1 Table, ID 15) also lacks a chemical analysis, but it is isotopically consistent with the ores from the Italian Alps in the region of Trentino-Bolzano (Fig 9).

The other flanged axe from Harpe, Saxony-Anhalt (S1 Table, ID 44) has a lead isotope composition similar to the axe from Ulvemosehusene in Denmark (S1 Table, ID 30) discussed above, and its chemical composition indicates that the copper was smelted from *Fahlerz* with a high content of antimony and silver. Perhaps incidentally, it displays a lead isotope composition identical to a sample from an ore deposit in Northwestern Italy (Piemonte, Val Sesia) published in a geo-chronological research paper [75] as well as the bornite minerals from Saint-Veran, in the French Alps [76]. However, there is no mention in the literature of the presence of *Fahlerz* in either of these two locations. In addition, even if the mines of Saint-Veran have been dated to the Early Bronze Age, no actual artefacts with these lead isotope ratios have been found yet in Italy or France ([77], 55). Nevertheless, the Western Alps appear at present to be the most probable source for the copper used to make this axe and, as discussed extensively by Artioli *et al*. [77] and Dolfini *et al*. [73], the copper ore deposits of Northwest Italy (Tuscany, Falda Piemontese, and other locations north of Torino and Milan) display great potential for future research into the early metallurgy of copper.

The two flat axes from Stolzenburg (S1 Table, ID 2) and Uelzen (S1 Table, ID 14) display so-called ‘radiogenic’ lead isotope ratios which are rather unusual among Bronze Age artefacts and usually only found among Early Bronze Age finds. There are four localities in Western Europe where such ores were mined in the Bronze Age: the Mitterberg in Austria [78], some mines in Northwestern Spain [79, 80] and the Guadalquivir Valley of Southern Spain [81], and the Great Orme in Wales [82].

**Fig 10:**
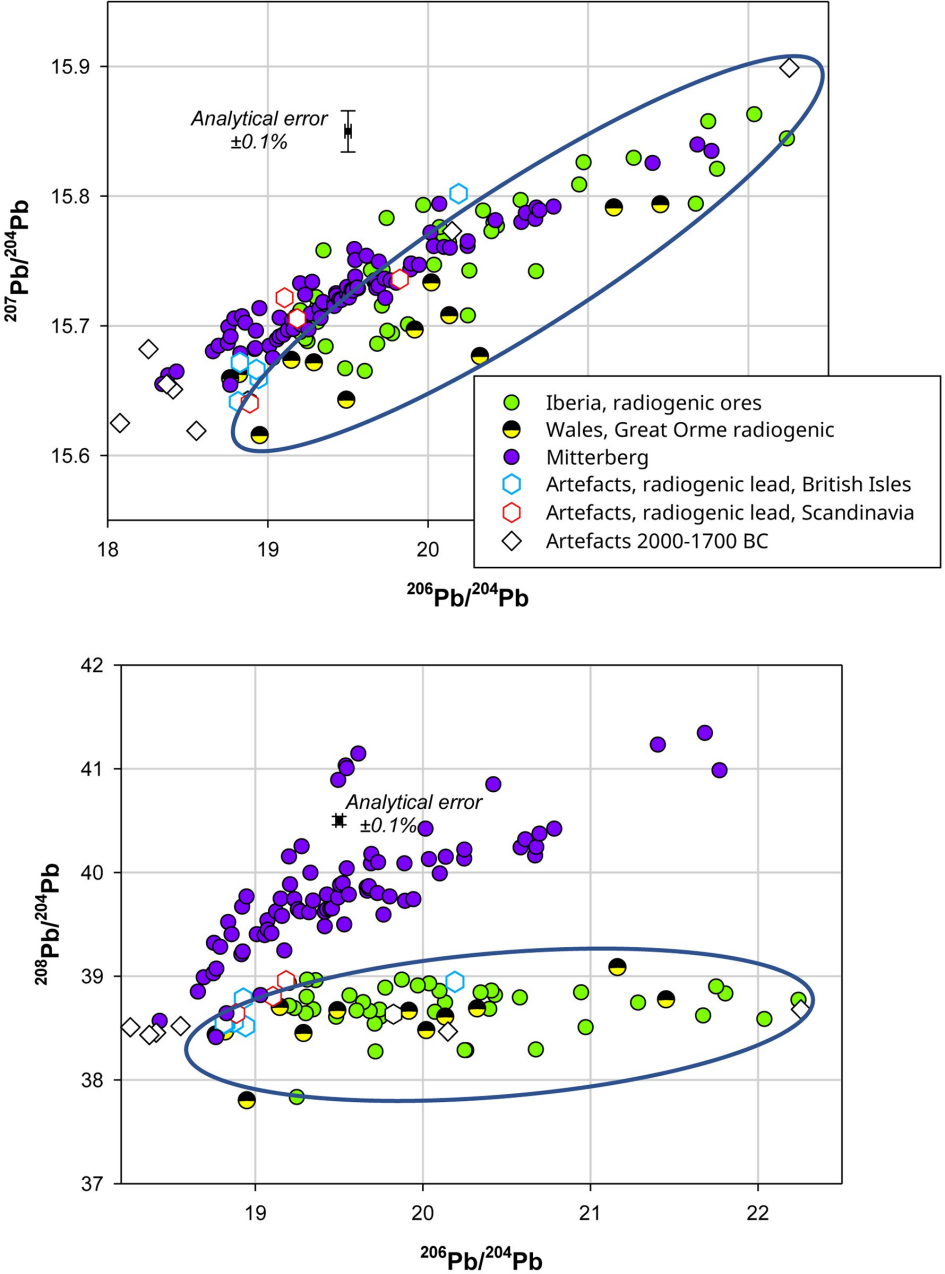
The radiogenic lead isotope ratios of the ores from Mitterberg [78], the Iberian Peninsula [43, 44] and the Great Orme mine of Wales [43, 83] compared with the two flat axes found in Germany and some copper artefacts from Britain [43] and Scandinavia [22, 32]. Blue ellipse is drawn to indicate clearly the difference between the lead isotope ratios of the ores from Mitterberg and the radiogenic lead in copper ores from the Great Orme mine of Wales and Iberia, it does not show the isotopic range of these ores.

**Fig 11:**
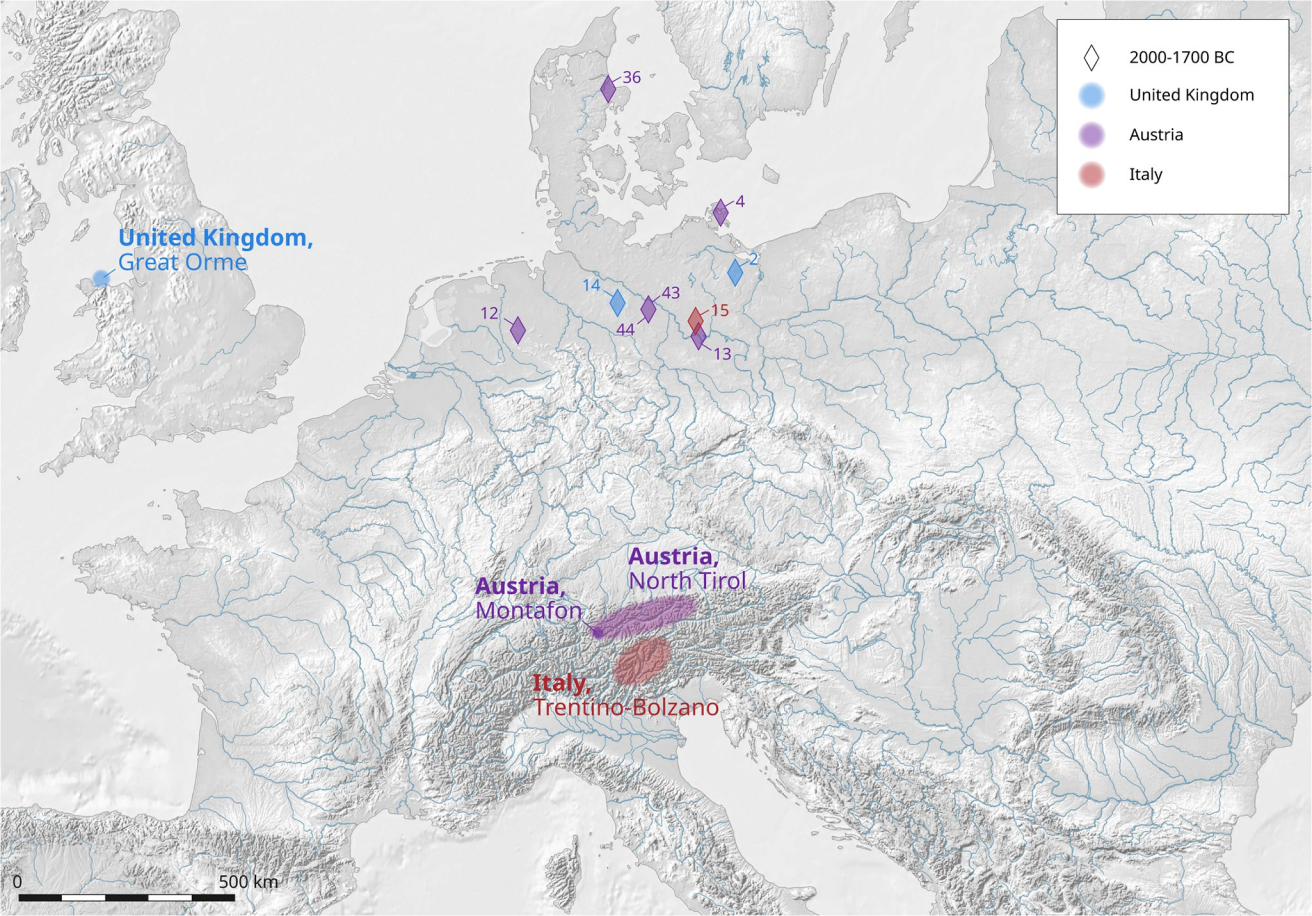
The sites of the sampled objects of Group 4 (ca. 2000–1700 BC) in the study area and the regions of origin of the copper (cf. S1 Table) based on the analyses of this study [IMAGINE TIFF support, copyright 1991–1999 by ERDAS, Inc. All Rights Reserved, @(#)$RCSfile: etif.c $ $Revision: 1.9.1.3 $ $Date: 2002/07/29 15:39:06EDT $] (C. Reckweg, CAU Kiel).

The lead isotope data for the deposits presented in Fig 10 clearly illustrates that only the ores from the Iberian Peninsula and the Great Orme are consistent with the ^208^Pb/^204^Pb ratios displayed by these two axes. In addition, there are several artefacts from Britain [61] and Scandinavia [22] which show some very similar lead isotope ratios. The lead isotope data for both of our axes is closer to the lead of the copper ores from the Guadalquivir Valley. However, the wide range of radiogenic ratios among the ores from the Great Orme does not preclude the possibility that these two axes could have been produced from the copper ores found in this location, in particular as their chemistry tallies rather well with the data published by Williams [82]. In addition, the period of activity of the Great Orme mine lies between 1850–900 BC ([82], 34, Fig 4) which could correspond to the 1950–1700 BC dating given for these two axes. Unless further evidence for the periods of exploitation and the geochemistry of the ores from the Iberian Peninsula and their use in Northern Europe is forthcoming, it would seem that the most probable origin of copper for these two axes is indeed the Great Orme mine of Wales (Figs 10 and 11).

## 4. Discussion—Copper in the context of the Neolithic and Bronze Age sphere

In the Northern Central European Plain and Southern Scandinavia, the intensity of agriculture and livestock breeding begins to increase significantly with the process of neolithisation from 4100/4000 BC onward [84–86]. At the same time, first the appearance and then the steady increase in the number of imported copper objects can be observed, contemporaneous with the introduction of other non-local objects [18]. This trend is also reflected in hoarding practices, for example, in Neuenkirchen (D) (earliest possible deposition around 3800/3700 BC) [46] and Bygholm (DK) (earliest possible deposition around 3600/3500 BC) [18]. In addition to the ability to mechanically process copper, pyrotechnic processing skills are also evident, as shown by a crucible and a possible nozzle from Lønt (DK), dating to a span from ca. 3800–3300 BC [28]. The deliberate fracture of the flat axe of Neuenkirchen through hot-shorting also suggests the presence of sound material and metallurgical knowledge as early as 3800/3700 BC [46]. The term hot shorting refers to the phenomenon that when a copper-alloy object is heated past a certain temperature, it causes great thermal stress within the material and it can fracture under pressure. The resulting separation into two pieces exhibits a clean, ‘sharp’ break which is accompanied only with limited deformation of the object or further fragmentation. This so-called ‘hot-shorting’ can happen by accident when quenching, working or deliberately destroying the object [87–89]. Both options have been observed in the archaeological record (ibid.).

Thus, in the north, the innovation of copper pyrotechnical processing seems to be introduced contemporaneously with similar evidence from Baalberge and Mondsee contexts from Southern Central Europe [90].

**Fig 12:**
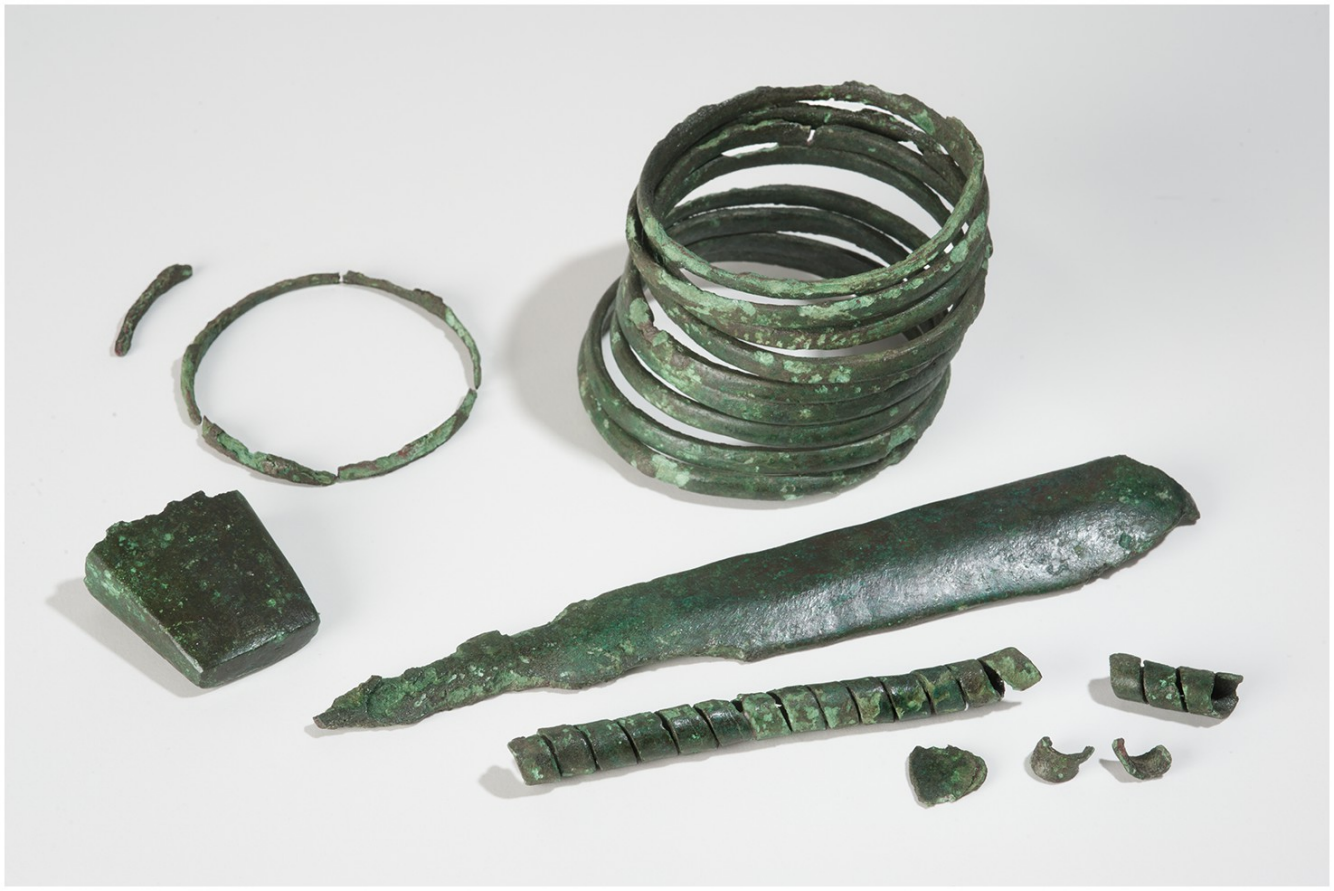
Inventory of the Neuenkirchen hoard (Photo A. Heitmann/H.Skorna, University of Kiel).

Within our data set, 10 flat axes and a Jászladány-type axe (S1 Table, ID 6, Karow) are from single find contexts and two depots and can be attributed to the period between 3750–3300 BC [18, 29, 47, 91, 92]. A similar dating is proposed for a flat axe from the Skudderup hoard (S1 Table, ID 27, Scania, Sweden). In addition, a flat axe (S1 Table, ID 42) from the above-mentioned Neuenkirchen hoard dates to an earliest possible deposition around 3800/3700 BC (Fig 12). For a flat axe of Kaka-type (generally dated between 4100 and 3400 BC) from Frömkenberg (S1 Table, ID 45), a date between 3800–3400 BC is assumed ([93], 56).

With regard to the regions of origin, the copper ores of the copper artefacts of our typo-chronological Group 1 (4100–3500 BC) derive from mines in Southeastern Europe, especially from the Serbian mining areas, namely the Majdanpek region of Serbia, and in one case (Pantelitz) the Burgas region of Bulgaria. This result is also supported by a recent study of Danish artefacts in which 10 flat axes of Bygholm type and two spirals from the period between 3800–2850 BC were examined. In this instance, Southeastern Europe (Serbian mining areas, in particular) could be established as the region of origin [4, 26]. Included are the flat axe and arm spiral from the Søby Hede hoard, a tongued-shaped flat axe from Viborg, the thick-butted axe from Slusegard, an axe from Moesgaard and the Bygholm flat axe from Kjelstrup. The last is interpreted to have derived from Bosnien ores [18, 26], while the others partly overlap with isotope signatures corresponding to the copper mines of Stara Zagora (Ai Bunar) and Burgas (Mendi Rid) and those of the East Serbian Copper Belt.

For our typo-chronological Group 2 (3500-3300/2800 BC), most of the analysed artefacts can be dated to ca. 3500–3300 BC. Further 15 flat axes as well as the three spirals (S1 Table, ID 17–19) and the flat axe (S1 Table, ID 16) from the Ratekau-Riesebusch date to these centuries.

The analysed spiral roll from the megalithic grave Emmeln (S1 Table, ID 20) and the flat axe from Herbede (S1 Table, ID 11) probably date to ca. 3300–3000 BC. Nevertheless, for the period between 3350-3000/2800 BC, copper artefacts can be discerned only in small numbers from the Northern Central European Plain and Southern Scandinavia [18, 94], as is also reflected in the small number objects analysed in our study. This contrasts with the much larger number of objects in the previous centuries and the accompanying knowledge of mechanical processing which had led to distinct types, as well as to the ability to pyrotechnically process copper starting at the latest at ca. 3800 BC.

The generally decreasing number of copper products or copper imports to the north from ca. 3300–2800 BC established by this study as well as others [18, 26] accords with a period of disintegration of copper supply networks between 3700–3200 BC in Southeastern Europe [95, 96]. Furthermore, during this period, other exclusive objects, such as jade axes, no longer found their way northward [97], with the exception of only a very few pieces [90].

However, this does not account for conditions in the northwestern part of Central Europe: For the period between 3300-3000/2900 BC, we know of a few copper finds (spirals) and a few copper shaft-hole axes. Copper spiral rolls were found in burials in the large megalithic tomb at Emmeln [49, 50], and the raw material of the one already mentioned spiral (S1 Table, ID 20) is assigned to the Slovakian copper mining areas. In addition, two spiral rings come from a megalithic tomb in the Netherlands ([18], 217–219). Even if no copper adzes are recorded for the 4^th^ millennium in the Netherlands [18], the mentioned flat axe from Herbede (S1 Table, ID 11) also belongs to this period. Furthermore, four copper shaft-hole axes do appear in the western part of the investigated area. The shape of these axes is similar to that of stone hammer axes (knob-butted [KIVb-type] and round-butted [RV-type] according to Zápotocký’s typology [98]) in the same region ([99], 251–255, cf. [98]). One copper axe (knob-butted type) belongs to the hoard from Osnabrück-Lüstringen, which also contains a large ring and two lunula pendants. A ^14^C dated cremation burial nearby (Beta-502565, 4430±30 BP [ca. 3330–2925 cal BC 2 sigm]; Poz-134314, 4240±35 BP [ca. 2900–2775 cal BC 2 sigm]), which is probably connected to the hoard, confirms the date ([99], 309, [100], 333). The assumed source of the material for the copper shaft-hole axes, which display a high arsenic content exceeding that of the originally defined “Mondsee copper” [101], is Southeastern Europe ([100], 333, [102], 325). Accordingly, the western part of the investigated area must have been characterised by a distinct supply network which connected this region with Southeastern Central and Eastern Europe, where similar knob-butted or related shaft-necked axes occur [99, 100, 103, 104].

All in all, the provenience of the copper raw material of the analysed items of Group 2 point, on the one hand, to the ores of the Eastern Alps, such as the Inn Valley of North Tyrol or Italy’s Trentino-Bolzano region and, on the other hand, to the Slovakian Ore Mountains. Furthermore, artefacts are isotopically consistent with the ores from Serbian deposits in the region of Majdanpek. Thus, the lead isotope analyses point to an integration of South Central European copper ores into the raw material supply, added to the still valid connectivity to the Southern Balkan supplies.

The described decline in the number of copper objects shows that the initial wave of copper imports and the adoption of metalworking had not led to what could be termed a point of no return. Instead, the centuries between ca. 3300 and 2800 BC, in particular, are characterised by an absence of imports, of the recycling of available metal, of the development of new types, and of the appearance of metal objects in alternative contexts such as burials. From this discontinuity, it can be concluded that copper objects must have been deliberately withdrawn from circulation during previous centuries, as suggested by the large number of individual finds of copper artefacts beforehand [18].

Collective burials, in contrast, are now marked by extensive grave goods, for example, highly decorated pottery vessels, accompanied by the agglomeration of people in villages and a general trend of demographic growth [105]. A form of cooperation seems to have determined the lives of people, at least according to outward appearances [106]. At the same time, flint axes and ground stone hammer axes were increasingly deposited in the landscape and in burials, obviously assuming the role of symbols of distinction in society [99, 107, 108]. In contrast to copper artefacts, these objects appear in greater numbers and in different contexts, and combine a strong symbolical character with a practical function as weapons.

This form of coexistence ends with the transition from the 4^th^ to the 3^rd^ millennium BC, a change which may be attributed to possible conflicts and reconciliations triggered by demographic growth and new cultural impulses [107, 108]. The latter include the appearance of the Corded Ware phenomenon in the form of the Single Grave society (SGC) [109] and the influences of the Globular Amphora phenomenon [110, 111], which engendered, among others, fundamental social changes.

With the transition from the 4^th^ to the 3^rd^ millennium BC and the following centuries, social differentiation was amplified in the SGC, a process characterised by battle axes, beakers, and single graves. Burial mounds and secondary burials show the emergence of a far-reaching social system, probably related to kinship, based on smaller groups and their trans-local relationships [109, 112–114]. In this context, the communication networks of the SGC groups would have become involved in the exchange of copper artefacts, albeit to a lesser extent. Quantitatively, objects are detectable only in small numbers ([18], 198–209, [94], 108–109).

The period between ca. 2800–2200 BC is represented in our study by an axe from Vietznitz (S1 Table, ID 1) made of material from the mining areas of Slovakia.

From 2300 BC onward, an increase in metallurgic activity can be observed. This includes the hoard from Pile in Scania, which can be dated to around 2000 BC [11]. In our research area, further indirect proof for metal processing–two cushion stones–comes from the Wakenitz River near Groß Sarau ([115], 316–317). The objects from our pilot study include the two flat axes from Ulvemosehusene (Zealand, DK, S1 Table, ID 30) and Flinterupgård (Zealand, DK, S1 Table, ID 32) dating to 2350–1950 BC, which might be made of copper from the Austrian Alps.

Three Zabitz-type double axes (S1 Table, ID 10, 21 and 22), dating probably ca. 2300–2000 BC, fit into the Slovakian ore group. According to Kibbert [47], Zabitz-type axes are dated to the late phase of the Final Neolithic and to the Early Bronze Age. This chronological assumption has to be variated [99]. The Zabitz-type is actually very heterogeneous. Kibbert [47] discerned three sub-types: Cochem, Flonheim, and Westeregeln. To these, the Bronze Age Dieskau-type double axes, such as those from the eponymous Dieskau hoard (ca. 2000–1700 BC) [116], can be added.

From a typological perspective, Cochem-type axes–large items that weigh up to 3.5 kg–closely resemble lithic double axes of the (late) Late Neolithic (Central European terminology) of the Alpine region, which are dendro-dated within the 29^th^-28^th^ century BC [99]. The general outline as well as the oval shaft holes are connecting features of lithic and copper axes. The copper double axes presumably date to the early 3^rd^ millennium BC [99, 116].

The three Zabitz-type axes addressed in this study belong to the Westeregeln sub-type. Typo-chronologically, this type can be placed between the Late Neolithic Cochem-type and the Bronze Age Dieskau-type axes [99]. The size and weight decrease in a significant manner from the supposedly early specimens to the late specimens. The supposedly early axes occur exclusively as single finds, sometimes from rivers [47]. The late Dieskau-type axes are often discovered in hoard contexts [116]. The Westeregeln axes occupy a typological position between the above types and are occasionally found in hoard contexts. A development is thus indicated in which the axes become smaller while the probability of ending up in multi-object hoards increases [99]. According to the results of the current study, the supposed typo-chronological progression from the Late Neolithic Cochem-type axes via the Westeregeln-type of Group 3 (2300–2000 BC) and finally to the Bronze Age Dieskau-type axes becomes much more likely. In summary, the artefacts in this group are produced with Slovakian or Austrian copper.

The last phase of the Neolithic, between ca. 1950–1700 BC (our Group 4), is represented in our analysis by five flat axes (S1 Table, ID 2, 4 and 13–15), three flanged axes from Harpe (S1 Table, ID 44), Leppin (S1 Table, ID 43) and Ahausen (S1 Table, ID 12), and one chisel from Assentoft (S1 Table, ID 36). Again, the raw material comes from different regions the Alps. The mining areas of Wales also constitute a potential source so that British ores come into play.

Within this phase, in general, the copper finds represent new networks and economic power. They reflect the extensive exchange relations between Southern Scandinavia, in low intensity up to Central Scandinavia, Western Europe with the British Isles, and Central Europe such as the Middle Elbe-Saale-Unstrut and the Polish Koscian region [11]. In addition to Alpine mines in our dataset, an intensification of exchange networks between Britain and Northern Central Europe and Southern Scandinavia occurs, visible with the hybrid British axes [26].

At the same time, a point of no return [5, 26] in the adaptation of metallurgy by northern societies is now being approached. This is certainly due to the renewed opportunities to display status within groups provided by the exotic objects, whereas technological advantages gained by the material used for tools can be excluded as a reason due to the low hardness of the material in the case of copper and the continued use of stone tools in the Nordic Bronze Age [117].

At the same time, economic growth, based on intensive agriculture, new crop species, and large-scale clearing of the landscape [84] can be observed. These developments are linked to participation in the extensive exchange networks that spread across the area between Southern and Central Scandinavia, the British Isles and the Únětice groups [11]. In the Late Neolithic, the size of longhouses [118, 119] increases in Southern Scandinavia, which might copy the huge Únětice houses and those of the Southern German Early Bronze Age ([120], [121], 243–245). Shortly thereafter (ca. 1800 BC), three-aisled houses occur in Jutland [122], whose predecessors also are to be found in the Únětice core-region. At the same time, new crops (*Triticum spelta*) arrive from Central Europe to the north ([123], 113, [124]), while human impact in Northern Germany can also be related to an expansion in economic intensity [125]. The exchange of goods is characterised by the export of amber and flint daggers of Type I to the south [126, 127]. The complexity of these networks is illustrated by the diversity of mines in our dataset as well as the local admixture of regionally different bronze artefact styles (British, Central German) into a Southern Scandinavian shape [11].

## 5. Conclusion

**Fig 13:**
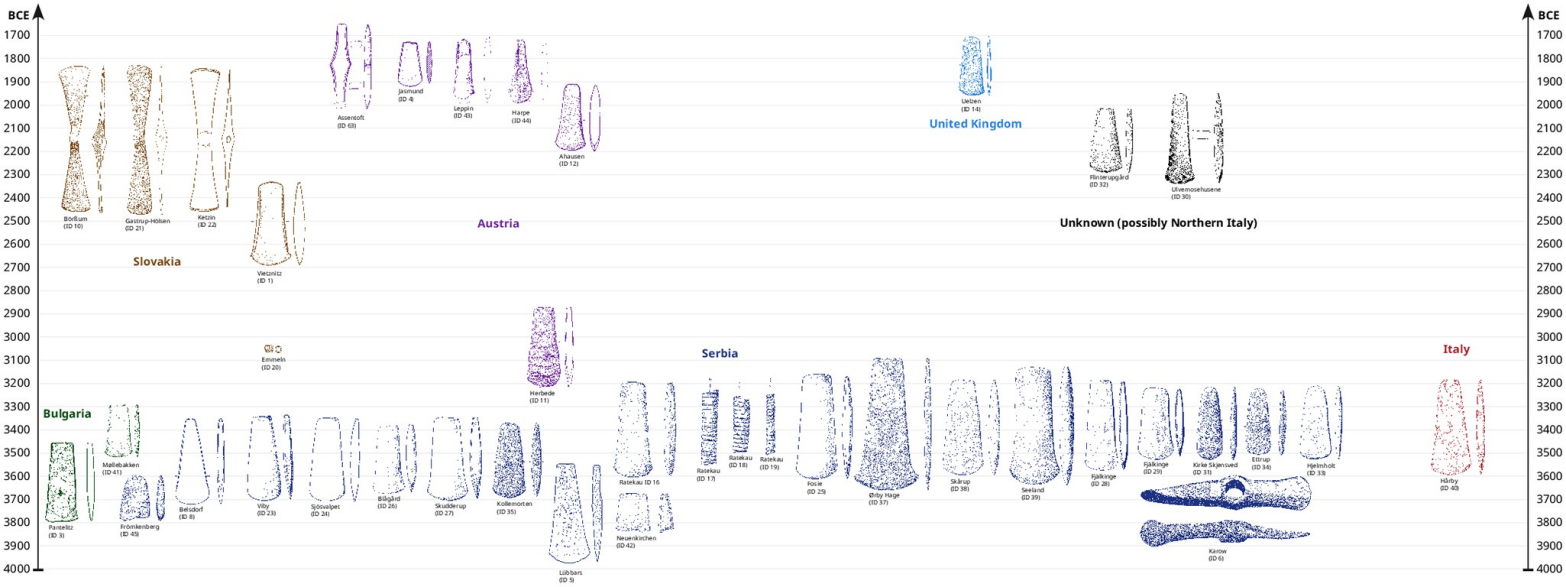
Chart of the chronological sequence of the Neolithic copper artefacts and the origin of the raw material in this study (C. Reckweg, CAU Kiel).

In summary, the new data displays changes within the raw material supply for Northern Central Europe and Southern Scandinavia. The beginning of copper imports at the beginning of the fourth millennium BC is linked to Southeast European copper ore deposits exclusively. This continued to be the case until ca. 3300 BC at the latest, especially during the peak of copper production (3500–3300 BC). At the earliest around 3300 BC, Eastern Alp and Slovak ore deposits were exploited. After the described decrease and gap in copper supplies between ca. 3300–2300 BC, from around 2300 BC onwards, only copper from South Central Europe, and from 2000 BC also from the British Isles, were used as raw material supplies (Fig 13).

While local metallurgical processing techniques were already present from 3800 BC at the latest, the role and meaning of copper artefacts was not the trigger of a social transformation of the northern societies. This phenomenon is also visible in the development of copper metallurgy in the Balkans (ca. 5000 BC). This also seems to have had very little impact on societies there, while the subsequent establishment of production and distribution of metal mainly influenced the identity of groups and individuals through common practices and cooperation. For the Vinča culture, in particular, an increase in wealth due to metallurgy is assumed, but other areas of life, such as the economy or demography, do not seem to have been affected to a higher degree [128]. The first integration of copper metallurgy and the use of copper objects in Neolithic societies in Northern Central Europe and Southern Scandinavia was therefore part of a wider development. This included communication and exchange networks as well as changes in the subsistence economy and new technologies, like the introduction of the ard [85] or wheel and wagon [129], as partial aspects of transformation processes in the 4^th^ millennium BC [130]. The early consolidation of copper metallurgy was not a sustainable process as a consequence, even if *sensu strictu* we are dealing with Chalcolithic societies. Not until the Late Neolithic and Early Bronze Age did Nordic societies integrate copper metallurgy in their economic system in such a way that these societies reached a point of no return with respect to metallurgy, increasingly using more metal artefacts as tools in the subsistence economy as well as for the display of power structures [11, 29, 30, 131].

## Supporting information

S1 Table Datasheet (Excel) of the lead isotope (MC-ICP-MS) and trace element (EDXRF) analyses executed on 45 artefacts. (XLSX)

S1 Data Figures of the analysed objects (C. Reckweg, CAU Kiel). (PDF)

S1 File (DOCX)
