# Supplementary material for: The origin of Neolithic copper on the central Northern European plain and in Southern Scandinavia: Connectivities on a European scale
Source: PLoS One. 2023 May 10;18(5):e0283007. doi: 10.1371/journal.pone.0283007 (PMC10171686; doi:10.1371/journal.pone.0283007)
Supplement: S1 Data — (PDF) [file pone.0283007.s002.pdf]

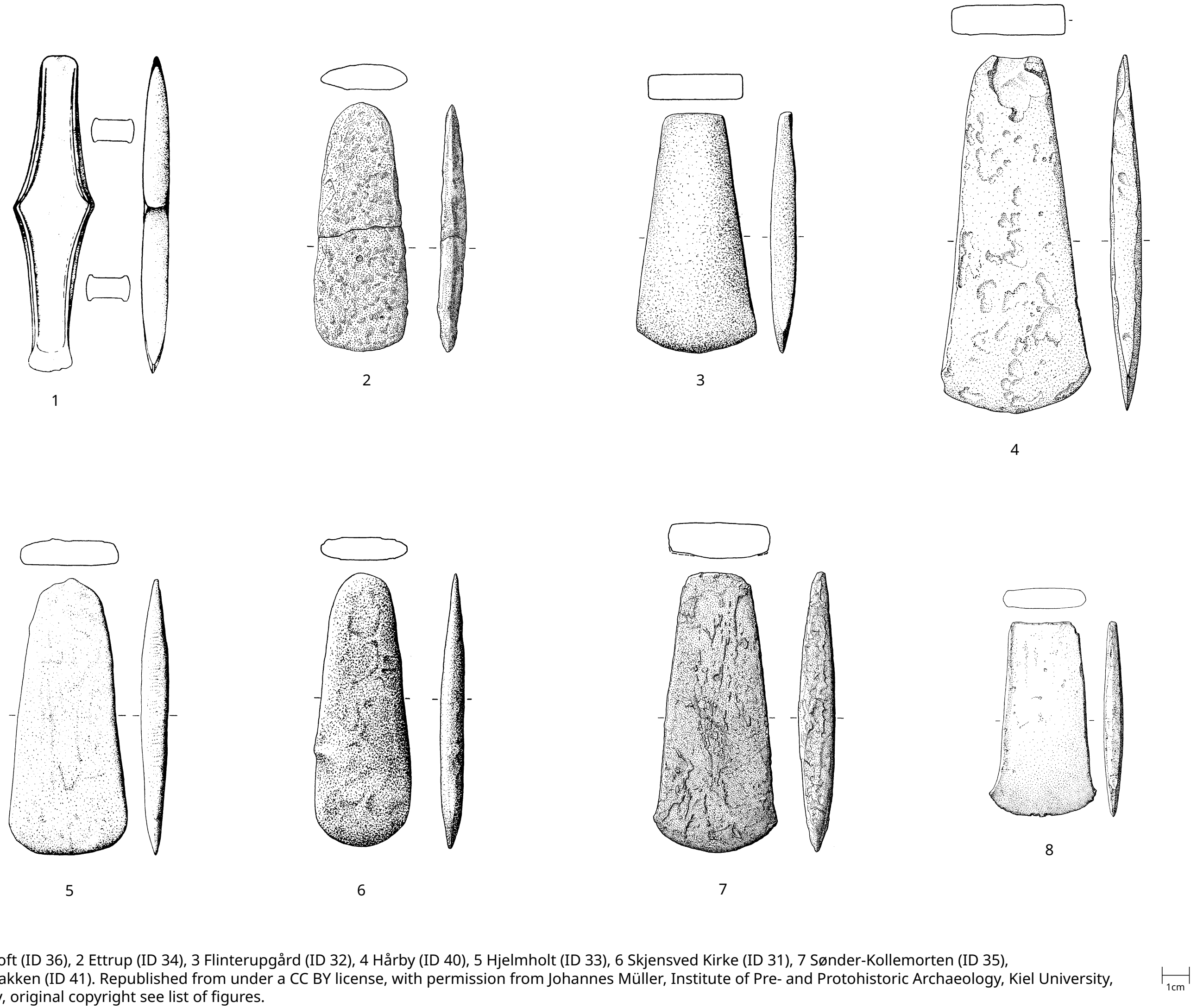

1 Assentoft (ID 36), 2 Ettrup (ID 34), 3 Flinterupgård (ID 32), 4 Hårby (ID 40), 5 Hjelmholt (ID 33), 6 Skjensved Kirke (ID 31), 7 Sønder-Kollemorten (ID 35), 8 Møllebakken (ID 41). Republished from under a CC BY license, with permission from Johannes Müller, Institute of Pre- and Protohistoric Archaeology, Kiel University, Germany, original copyright see list of figures.

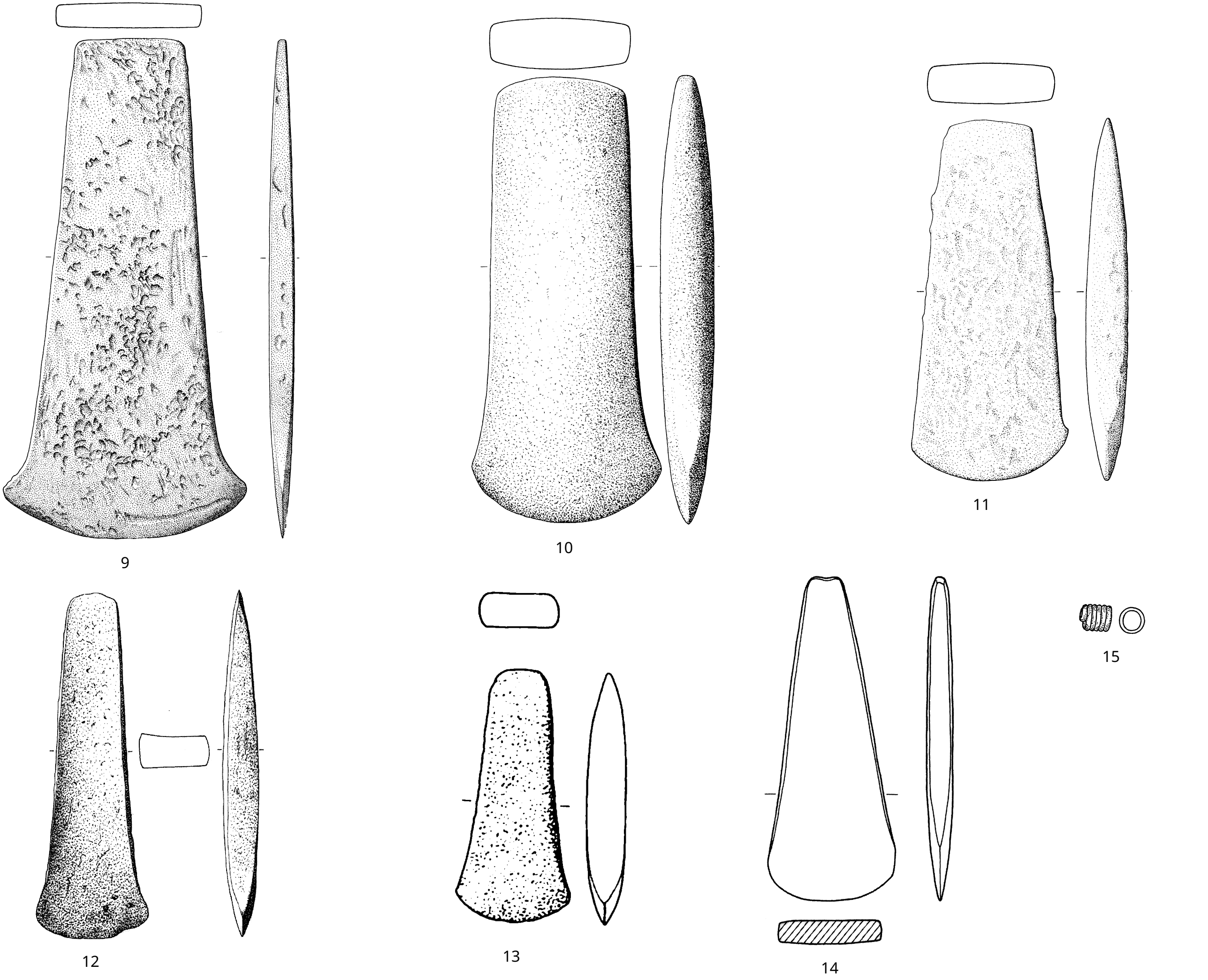

9 Ørby Hage (ID 37), 10 Seeland (ID 39), 11 Skårup (ID 38), 12 Ulvemosehusene (ID 30), 13 Ahausen (ID 12), 14 Belsdorf (ID 8), 15 Emmeln (ID 20). Republished from under a CC BY license, with permission from Johannes Müller, Institute of Pre- and Protohistoric Archaeology, Kiel University, Germany, original copyright see list of figures.

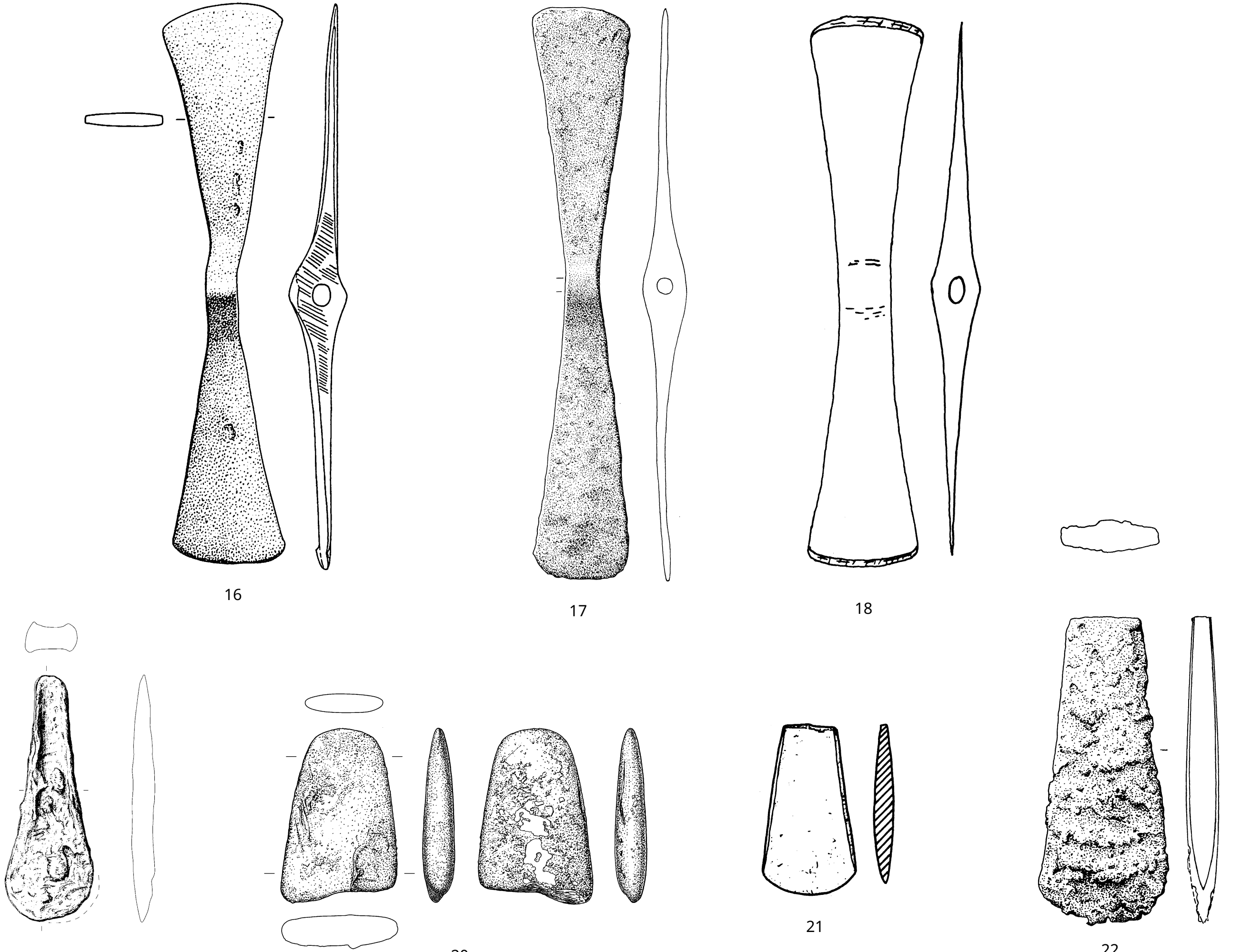

16 Börßum (ID 10), 17 Gastrup-Hölsen (ID 21), 18 Ketzin (ID 22), 19 Harpe (ID 44), 20 Frömkenberg (ID 45), 21 Jasmund (ID 4), 22 Herbede (ID 11). Figures 16-18 and 20-22: Republished from under a CC BY license, with permission from Johannes Müller, Institute of Pre- and Protohistoric Archaeology, Kiel University, Germany, original copyright see list of figures. Figure 19: Republished from under a CC BY license, with permission from Barbara Fritsch, Landesamt für Denkmalpflege und Archäologie Halle (Saale), Germany, original copyright see list of figures.

1cm

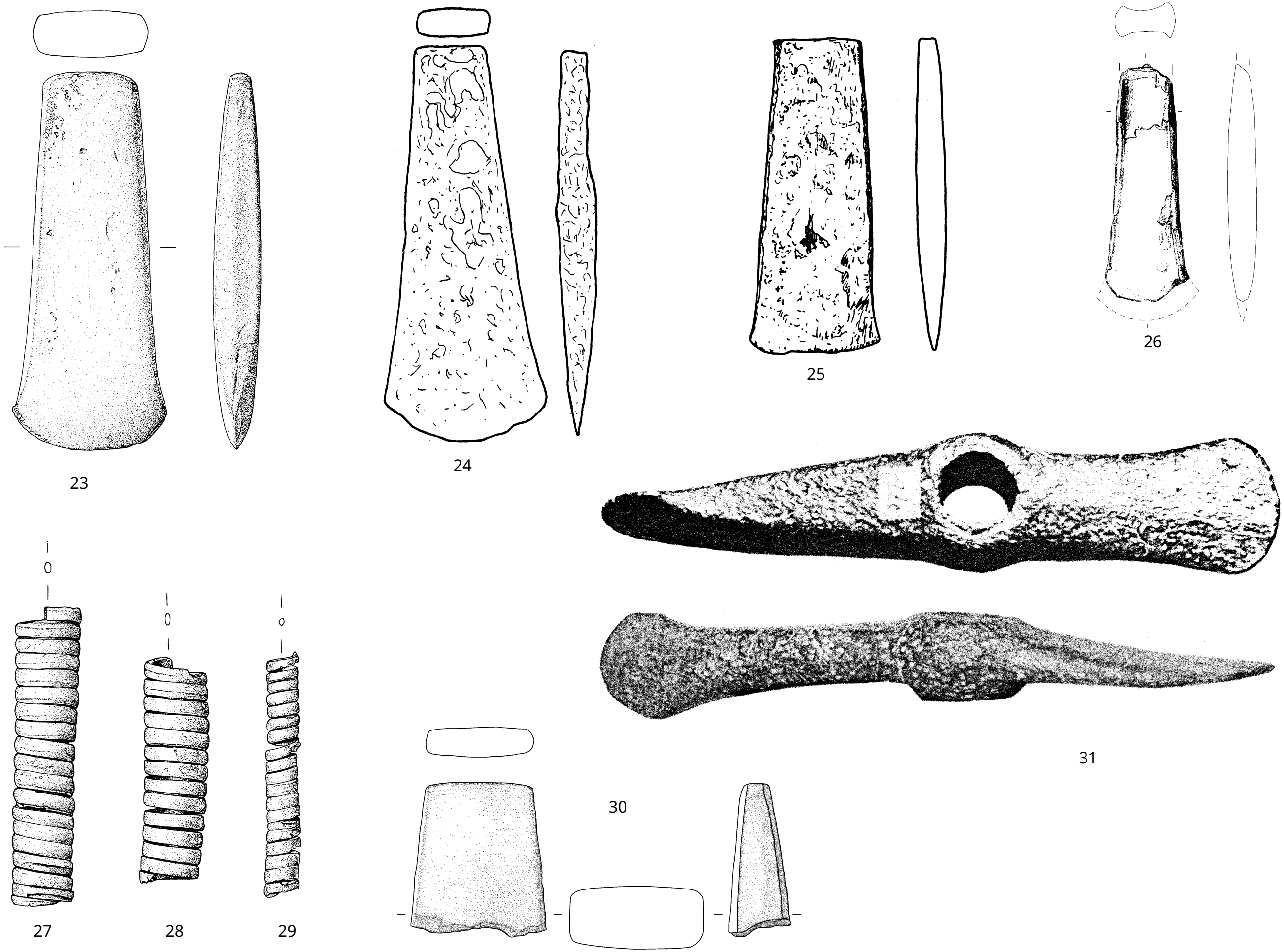

23 Ratekau (ID 16), 24 Lübbars (ID 5), 25 Pantelitz (ID 3), 26 Leppin (ID 43), 27 Ratekau (ID 17), 28 Ratekau (ID 18), 29 Ratekau (ID 19), 30 Neuenkirchen (ID 42), 31 Karow (ID 6). Figures 23, 25, 27-31: Republished from under a CC BY license, with permission from Johannes Müller, Institute of Pre- and Protohistoric Archaeology, Kiel University, Germany, original copyright see list of figures. Figures 24 and 26: Republished from under a CC BY license, with permission from Barbara Fritsch, Landesamt für Denkmalpflege und Archäologie Halle (Saale), Germany, original copyright see list of figures.

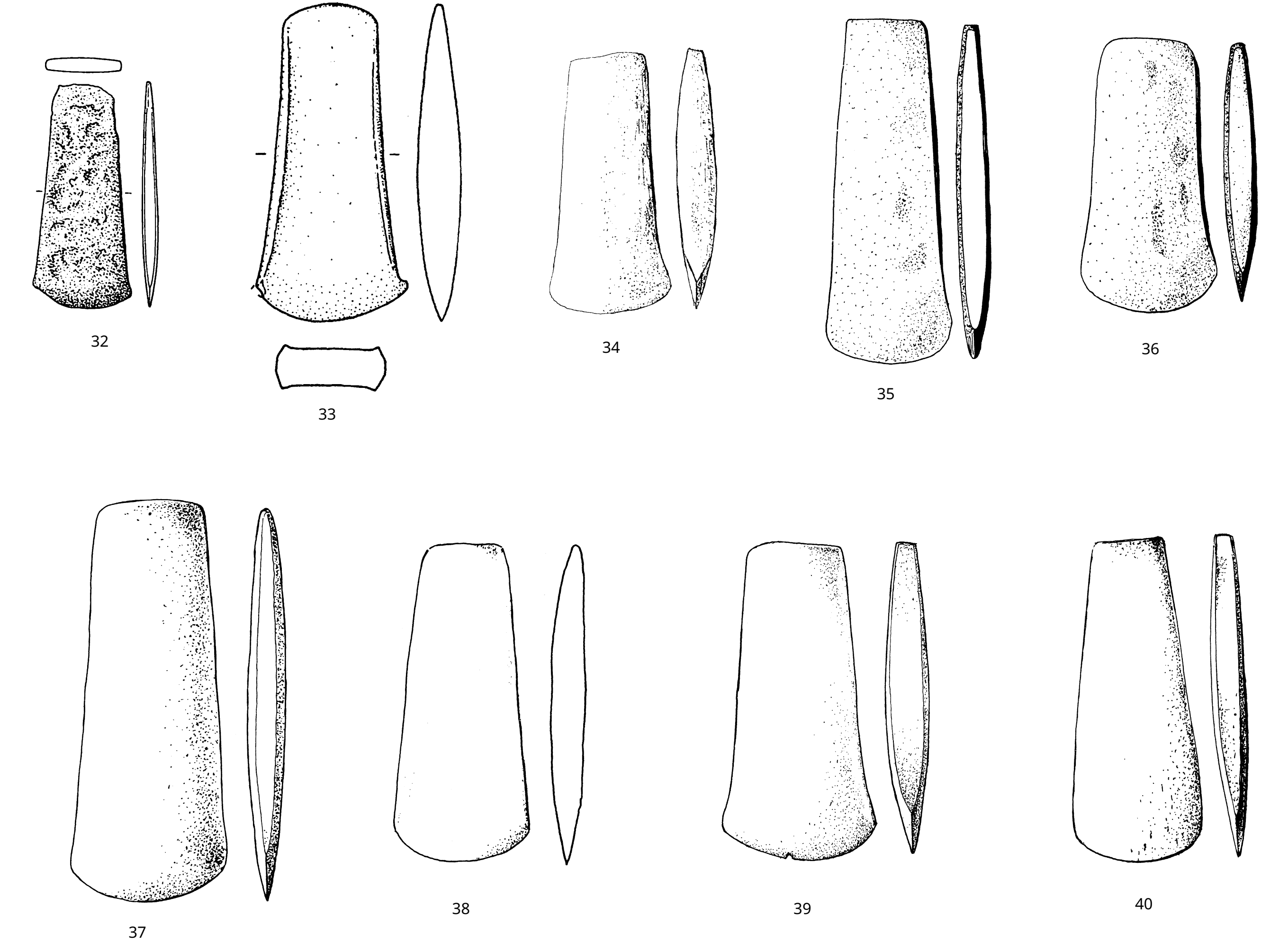

32 Uelzen (ID 14), 33 Vietznitz (ID 1), 34 Blågård (ID 26), 35 Fjälkinge (ID 28), 36 Fjälkinge (ID 29), 37 Fosie (ID 25), 38 Sjösvalpet (ID 24), 39 Skudderup (ID 27), 40 Viby (ID 23). Republished from under a CC BY license, with permission from Johannes Müller, Institute of Pre- and Protohistoric Archaeology, Kiel University, Germany, original copyright see list of figures.

1cm

List of figures

1 Assentoft (ID 36)
Vandkilde H. From stone to bronze: the Metalwork of the late Neolithic and Earliest Bronze Age in Denmark. Jutland Archaeological Society publications 32. Aarhus: Aarhus University Press; 1996. p. 135, fig. 129.

2 Ettrup (ID 34)
Klassen L. Frühes Kupfer im Norden. Untersuchungen zur Chronologie, Herkunft und Bedeutung der Kupferfunde der Nordgruppe der Trichterbecherkultur. Jutland Archaeological Society publications 36. Aarhus: Aarhus University Press; 2000. German. Plate 3.12.

3 Flinterupgård (ID 32)
Klassen L. Frühes Kupfer im Norden. Untersuchungen zur Chronologie, Herkunft und Bedeutung der Kupferfunde der Nordgruppe der Trichterbecherkultur. Jutland Archaeological Society publications 36. Aarhus: Aarhus University Press; 2000. German. Plate 4.18.

4 Hårby (ID 40)
Klassen L. Frühes Kupfer im Norden. Untersuchungen zur Chronologie, Herkunft und Bedeutung der Kupferfunde der Nordgruppe der Trichterbecherkultur. Jutland Archaeological Society publications 36. Aarhus: Aarhus University Press; 2000. German. Plate 13.51.

5 Hjelmholt (ID 33)
Klassen L. Frühes Kupfer im Norden. Untersuchungen zur Chronologie, Herkunft und Bedeutung der Kupferfunde der Nordgruppe der Trichterbecherkultur. Jutland Archaeological Society publications 36. Aarhus: Aarhus University Press; 2000. German. Plate 2.7.

6 Kirke Skjensved (ID 31)
Klassen L. Frühes Kupfer im Norden. Untersuchungen zur Chronologie, Herkunft und Bedeutung der Kupferfunde der Nordgruppe der Trichterbecherkultur. Jutland Archaeological Society publications 36. Aarhus: Aarhus University Press; 2000. German. Plate 2.9.

7 Sønder-Kollemorten (ID 35)
Klassen L. Frühes Kupfer im Norden. Untersuchungen zur Chronologie, Herkunft und Bedeutung der Kupferfunde der Nordgruppe der Trichterbecherkultur. Jutland Archaeological Society publications 36. Aarhus: Aarhus University Press; 2000. German. Plate 5.22.

8 Møllebakken (ID 41)
Klassen L. Frühes Kupfer im Norden. Untersuchungen zur Chronologie, Herkunft und Bedeutung der Kupferfunde der Nordgruppe der Trichterbecherkultur. Jutland Archaeological Society publications 36. Aarhus: Aarhus University Press; 2000. German. Plate 13.52.

9 Ørby Hage (ID 37)
Klassen L. Frühes Kupfer im Norden. Untersuchungen zur Chronologie, Herkunft und Bedeutung der Kupferfunde der Nordgruppe der Trichterbecherkultur. Jutland Archaeological Society publications 36. Aarhus: Aarhus University Press; 2000. German. Plate 5.21.

10 Seeland (ID 39)
Klassen L. Frühes Kupfer im Norden. Untersuchungen zur Chronologie, Herkunft und Bedeutung der Kupferfunde der Nordgruppe der Trichterbecherkultur. Jutland Archaeological Society publications 36. Aarhus: Aarhus University Press; 2000. German. Plate 3.16.

11 Skårup (ID 38)
Klassen L. Frühes Kupfer im Norden. Untersuchungen zur Chronologie, Herkunft und Bedeutung der Kupferfunde der Nordgruppe der Trichterbecherkultur. Jutland Archaeological Society publications 36. Aarhus: Aarhus University Press; 2000. German. Plate 4.20.

12 Ulvemosehusene (ID 30)
Vandkilde H. From stone to bronze: the Metalwork of the late Neolithic and Earliest Bronze Age in Denmark. Jutland Archaeological Society publications 32. Aarhus: Aarhus University Press; 1996. p. 56, fig. 29.1.

13 Ahausen (ID 12)
Laux F. Die Äxte und Beile in Niedersachsen. Stuttgart: Steiner, 2000. German. Plate 10.155.

14 Belsdorf (ID 8)
Klassen K, Dobeš M, Pétrequin P. Dreieckige Kupferflachbeile aus Mitteldeutschland und Böhmen. Alt-Thüringen. 2008/2009, 41: 7-35. German. p. 16, fig. 8.2.

15 Emmeln (ID 20)
Schlicht E. Die Funde aus dem Megalithgrab 2 von Emmeln, Kreis Meppen: Studien zur Keramik der Trichterbecherkultur im Gebiet zwischen Weser und Zuidersee. Neumünster: Wachholtz; 1968. German. p. 19, fig. 4.a.

16 Börßum (ID 10)
Laux F. Die Äxte und Beile in Niedersachsen. Stuttgart: Steiner, 2000. German. Plate 71.1055.

17 Gastrup-Hölsen (ID 21)
Kibbert K. Die Äxte und Beile im mittleren Westdeutschland. München: Beck; 1980. German. Plate 4.16.

18 Ketzin (ID 22)
Jacobs J. Jungsteinzeitliche Metallfunde auf dem Gebiet der DDR. Zeitschrift für Archäologie. 1989: 23: 1-17. German. p. 10, fig. 4.2.

19 Harpe (ID 44)
Wiegmann M. Halle (Saale).

20 Frömkenberg (ID 45)
Jürgens F, Szillus C. Das älteste Metall in Ostwestfalen. Ein neues Kupferbeil aus Willebadessen-Peckelsheim. In: LWL-Archäologie für Westfalen, Altertumskommission für Westfalen, editors. Archäologie in Westfalen-Lippe 2018 (2019). Langenweißbach: Beier & Beran; 2019. pp. 55–58. doi: 10.11588/aiw.2019.0.76840. German. p. 56, fig. 2.

21 Jasmund (ID 4)
Kersten K. Die Funde der älteren Bronzezeit in Pommern. Hamburg: Hamburgisches Museum für Vökerkunde und Vorgeschichte; 1958. German. Plate 4.43.

22 Herbede (ID 11)
Kibbert K. Die Äxte und Beile im mittleren Westdeutschland. München: Beck; 1980. German. Plate 5.32.

23 Ratekau (ID 16)
Klassen L. Frühes Kupfer im Norden. Untersuchungen zur Chronologie, Herkunft und Bedeutung der Kupferfunde der Nordgruppe der Trichterbecherkultur. Jutland Archaeological Society publications 36. Aarhus: Aarhus University Press; 2000. German. Plate 26.98 A.

24 Lübbars (ID 5)
Stephan E. Die ältere Bronzezeit in der Altmark. Halle (Saale): Niemeyer; 1956. German. Plate VII.1.

25 Pantelitz (ID 3)
Kersten K. Die Funde der älteren Bronzezeit in Pommern. Hamburg: Hamburgisches Museum für Völkerkunde und Vorgeschichte; 1958. German. Plate 19.245.

26 Leppin (ID 43)
Wiegmann M. Halle (Saale).

27 Ratekau (ID 17)
Klassen L. Frühes Kupfer im Norden. Untersuchungen zur Chronologie, Herkunft und Bedeutung der Kupferfunde der Nordgruppe der Trichterbecherkultur. Jutland Archaeological Society publications 36. Aarhus: Aarhus University Press; 2000. German. Plate 26.98 D.

28 Ratekau (ID 18)
Klassen L. Frühes Kupfer im Norden. Untersuchungen zur Chronologie, Herkunft und Bedeutung der Kupferfunde der Nordgruppe der Trichterbecherkultur. Jutland Archaeological Society publications 36. Aarhus: Aarhus University Press; 2000. German. Plate 26.98 E.

29 Ratekau (ID 19)
Klassen L. Frühes Kupfer im Norden. Untersuchungen zur Chronologie, Herkunft und Bedeutung der Kupferfunde der Nordgruppe der Trichterbecherkultur. Jutland Archaeological Society publications 36. Aarhus: Aarhus University Press; 2000. German. Plate 26.98 F.

30 Neuenkirchen (ID 42)
Skorna H. The life and journey of neolithic copper objects. Transformations of the Neuenkirchen Hoard, North-East Germany (3800 BCE). Kirleis W, Müller J, editors. Scales of Transformation in prehistoric and archaic societies 15. Leiden: Sidestone Press; Forthcoming 2022. Plate 2.a.

31 Karow (ID 6)
Mötefindt H. Ungarische Doppeläxte aus der Provinz Sachsen. Jahresschrift für die Vorgeschichte der sächsisch-thüringischen Länder. 1911, 10: 73-76. German. Plate X.1a-b.

32 Uelzen (ID 14)
Laux F. Die Äxte und Beile in Niedersachsen. Stuttgart: Steiner, 2000. German. Plate 1.3.

33 Vietznitz (ID 1)
Lutz J, Matuschik I, Pernicka E, Rassmann K. Die frühesten Metallfunde in Mecklenburg-Vorpommern im Lichte neuer Metallanalysen. Vom Endmesolithikum bis zur frühen Bronzezeit. Bodendenkmalpflege in Mecklenburg-Vorpommern. 1997; 45: 41–67. German. Plate 2.9.

34 Blågård (ID 26)
Klassen L. Frühes Kupfer im Norden. Untersuchungen zur Chronologie, Herkunft und Bedeutung der Kupferfunde der Nordgruppe der Trichterbecherkultur. Jutland Archaeological Society publications 36. Aarhus: Aarhus University Press; 2000. German. Plate 16.67.

35 Fjälkinge (ID 28)
Klassen L. Frühes Kupfer im Norden. Untersuchungen zur Chronologie, Herkunft und Bedeutung der Kupferfunde der Nordgruppe der Trichterbecherkultur. Jutland Archaeological Society publications 36. Aarhus: Aarhus University Press; 2000. German. Plate 27.99 A.

36 Fjälkinge (ID 29)
Klassen L. Frühes Kupfer im Norden. Untersuchungen zur Chronologie, Herkunft und Bedeutung der Kupferfunde der Nordgruppe der Trichterbecherkultur. Jutland Archaeological Society publications 36. Aarhus: Aarhus University Press; 2000. German. Plate 27.99 B.

37 Fossie (ID 25)
Klassen L. Frühes Kupfer im Norden. Untersuchungen zur Chronologie, Herkunft und Bedeutung der Kupferfunde der Nordgruppe der Trichterbecherkultur. Jutland Archaeological Society publications 36. Aarhus: Aarhus University Press; 2000. German. Plate 18.76.

38 Sjösvalpet (ID 24)
Klassen L. Frühes Kupfer im Norden. Untersuchungen zur Chronologie, Herkunft und Bedeutung der Kupferfunde der Nordgruppe der Trichterbecherkultur. Jutland Archaeological Society publications 36. Aarhus: Aarhus University Press; 2000. German. Plate 17.71.

39 Skudderup (ID 27)
Klassen L. Frühes Kupfer im Norden. Untersuchungen zur Chronologie, Herkunft und Bedeutung der Kupferfunde der Nordgruppe der Trichterbecherkultur. Jutland Archaeological Society publications 36. Aarhus: Aarhus University Press; 2000. German. Plate 16.69.

40 Viby (ID 23)
Klassen L. Frühes Kupfer im Norden. Untersuchungen zur Chronologie, Herkunft und Bedeutung der Kupferfunde der Nordgruppe der Trichterbecherkultur. Jutland Archaeological Society publications 36. Aarhus: Aarhus University Press; 2000. German. Plate 16.66.
