# Supplementary material for: The origin of Neolithic copper on the central Northern European plain and in Southern Scandinavia: Connectivities on a European scale
Source: PLoS One. 2023 May 10;18(5):e0283007. doi: 10.1371/journal.pone.0283007 (PMC10171686; doi:10.1371/journal.pone.0283007)
Supplement: S1 File — (DOCX) [file pone.0283007.s003.docx]

04.03.2023

Dear PLOS ONE staff,

The permission to republish the graphics, with the exception of graphics 19, 24 and 26, in S2 plates is given by Johannes Müller, Institute for Pre- and Protohistoric Archaeology, Kiel University, Kiel, Germany.

The permission to republish the graphics 19, 24 and 26 in S2 plates is given by Barbara Fritsch, Landesamt für Denkmalpflege und Archäologie Halle (Saale), Germany,
